# MYCBP interacts with Sakura and Otu and is essential for germline stem cell renewal and differentiation and oogenesis

**DOI:** 10.1371/journal.pgen.1011792

**Published:** 2025-12-01

**Authors:** Azali Azlan, Ryuya Fukunaga

**Affiliations:** Department of Cell Biology, Johns Hopkins School of Medicine, Baltimore, Maryland, United States of America; Peter MacCallum Cancer Institute Central Cancer Library: Peter MacCallum Cancer Centre, AUSTRALIA

## Abstract

The self-renewal and differentiation of germline stem cells (GSCs) are tightly regulated during oogenesis. The *Drosophila* female germline provides a powerful model to study these regulatory mechanisms. We previously identified Sakura (also known as Bourbon/CG14545) as a crucial factor for maintenance and differentiation of GSCs and oogenesis, and demonstrated that Sakura binds to Ovarian Tumor (Otu), another essential regulator of these processes. Here, we identify MYCBP (c-Myc binding protein) as an additional essential component of this regulatory network. We show that MYCBP physically associates with itself, Sakura, and Otu, forming binary and ternary complexes including a MYCBP•Sakura•Otu complex. MYCBP is highly expressed in the ovary, and *mycbp* null mutant females exhibit rudimentary ovaries with germline-less and tumorous ovarioles, fail to produce eggs, and are completely sterile. Germline-specific depletion of *mycbp* disrupts Dpp/BMP signaling, causing aberrant expression of *bag-of-marbles* (*bam*) and leading to defective differentiation and GSC loss. In addition, *mycbp* is required for female-specific splicing of *sex-lethal* (*sxl*), a master regulator of sex identity determination. These phenotypes closely resemble those observed in *sakura* and *otu* mutants. Together, our findings reveal that MYCBP functions in concert with Sakura and Otu to coordinate self-renewal and differentiation of GSCs and oogenesis in *Drosophila*.

## Author summary

Stem cells are unique because they can both renew themselves and generate specialized cells, ensuring lifelong tissue maintenance and reproduction. In the fruit fly *Drosophila melanogaster*, germline stem cells (GSCs) in the ovary produce eggs, making this system a powerful model to understand how stem cells are regulated. We previously identified a female-germline protein, Sakura, that partners with another factor, Ovarian Tumor (Otu), to control germline stem cell behavior and oogenesis. In this study, we discovered that a third protein, MYCBP, works together with Sakura and Otu to form a regulatory ternary complex that is essential for oogenesis. Female flies lacking MYCBP cannot produce eggs, and their ovaries show both germline stem cell loss and uncontrolled germline stem cell overgrowth. We also found that MYCBP is required for proper signaling pathways that balance stem cell self-renewal and differentiation, suppression of harmful transposons, and the female-specific splicing of the master sex regulator gene, *sex-lethal*. These defects closely resemble those in *sakura* and *otu* mutants, highlighting that the three proteins act in concert. Because MYCBP is evolutionarily conserved including in human, our findings provide new insights into fundamental mechanisms of stem cell regulation and oogenesis that may extend beyond flies.

## Introduction

Oogenesis—the process by which germline stem cells (GSCs) develop into mature female gametes (oocytes)—is governed by multiple layers of regulation involving numerous genes. GSCs maintain their undifferentiated state while undergoing asymmetric division to produce one self-renewing GSC and one differentiating daughter cell, known as a cystoblast. Disruption of this balance can lead to either GSC loss, impairing oocyte production, or the overproliferation of undifferentiated cells, resulting in tumorous phenotypes and compromised fertility [1,2,3,4]. While precise control of GSC self-renewal and cystoblast differentiation is essential for proper oogenesis, the underlying molecular mechanisms remain incompletely understood, and additional regulatory factors likely remain to be identified.

**Fig 1:**
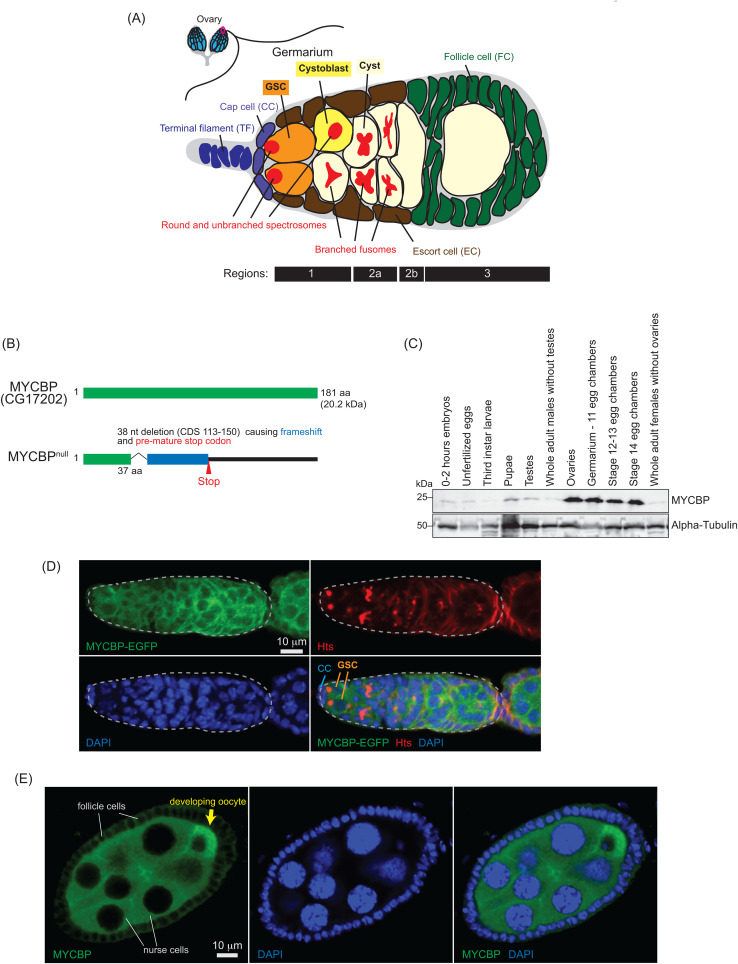
MYCBP expression pattern and mutant allele. **(A)** Schematic illustration of *Drosophila* ovary and germarium. Each female has a pair of ovaries, each consisting of 12-16 ovarioles (cyan). The germarium (outlined in magenta) is located at the anterior tip of each ovariole and contains both germ cells and somatic cells. Germ cells include germline stem cells (GSCs), cystoblasts, cysts, and differentiating oocytes. Somatic cells include terminal filament (TF) cells, cap cells (CCc), escort cells (ECs), and follicle cells (FCs). GSCs and cystoblasts have spherical, unbranched spectrosomes, whereas cysts posesss branched fusomes. The distinct regions of the germarium—1, 2a, 2b, and 3—are indicated. This is the same figure as we used in our previous publication [8]. **(B)** *Drosophila* MYCBP (CG17202) protein and the null mutant allele generated in this study. **(C)** Western blot of dissected fly tissues. **(D)** Confocal images of germaria from *mycbp-EGFP* transgenic flies. MYCBP-EGFP (green), Hts (red), and DAPI (blue). Scale bar: 10 μm. **(E)** Confocal images of egg chambers from *w1118* flies. MYCBP (green) and DAPI (blue). MYCBP is expressed in nurse cells, enriched in the developing oocyte (yellow arrow), and is also lowly detectable in somatic follicle cells. Scale bar: 10 μm.

The fruit fly *Drosophila melanogaster*, a genetically tractable model organism, has long served as a powerful system for studying GSC regulation and and oogenesis research [5]. *Drosophila* females possess a pair of ovaries, each composed of 12–16 ovarioles. At the anterior tip of each ovariole is a structure known as the germarium, which typically houses two to three GSCs (Fig 1A). These GSCs reside at the anterior-most region, in direct contact with cap cells and escort cells that form a specialized niche required for GSC maintenance [6,7]. Upon asymmetric mitotic division, a GSC produces a daughter GSC and a cystoblast, which subsequently undergoes four rounds of mitotic divisions with incomplete cytokinesis to form a 16-cell cyst. Of these 16 interconnected cells, one becomes the oocyte, and the remaining 15 differentiate into polyploid nurse cells that supply the oocyte with essential RNAs and proteins.

We recently identified a novel gene, *sakura* (also known as *bourbon/CG14545*) as an essential factor for GSC self-renewal and differentiation and oogenesis in Drosophila [8]. The Sakura protein, consisting of 114 amino acids, is exclusively expressed in female germline cells, including GSCs. We found that Sakura physically interacts with Ovarian Tumor (Otu), a known regulator of these same processes. Mutations in either *sakura* or *otu* result in tumorous germline overgrowth, germ cell loss, defects in oocyte specification, and aberrations in sexual identity determination, including the failure of female-specific splicing of *sex-lethal* (*sxl*) mRNA and the consequent production of the male-specific isoform [8–17]. Otu is known to form a deubiquitinase complex with Bag-of-marbles (Bam), a key differentiation factor, and to deubiquitinate Cyclin A (CycA), thereby, stabilizing CycA and promoting GSC differentiation [18]. In addition, Otu has been shown to bind RNA [19]. However, the detailed molecular mechanisms by which Otu and Sakura function in GSC self-renewal and differentiation and oogenesis remain poorly understood.

In this study, we identify c-Myc binding protein (MYCBP, CG17202) (Fig 1B), an uncharacterized 181-amino-acid protein and the *Drosophila* ortholog of human c-Myc-binding protein, as a critical factor for GSC self-renewal and differentiation and oogenesis. We show that MYCBP physically associates with itself, Sakura, and Otu, forming binary and ternary complexes including a MYCBP•Sakura•Otu complex. MYCBP is highly expressed in female germline cells and is required for GSC self-renewal and differentiation, oogenesis, and female-specific splicing of *sxl* mRNA. Strikingly, *mycbp*, *sakura*, and *otu* mutants exhibit similar phenotypes. Our findings suggest that MYCBP functions together with Sakura and Otu as a critical module governing GSC regulation and oogenesis.

## Results

### MYCBP is highly expressed in the ovaries

**Fig 2:**
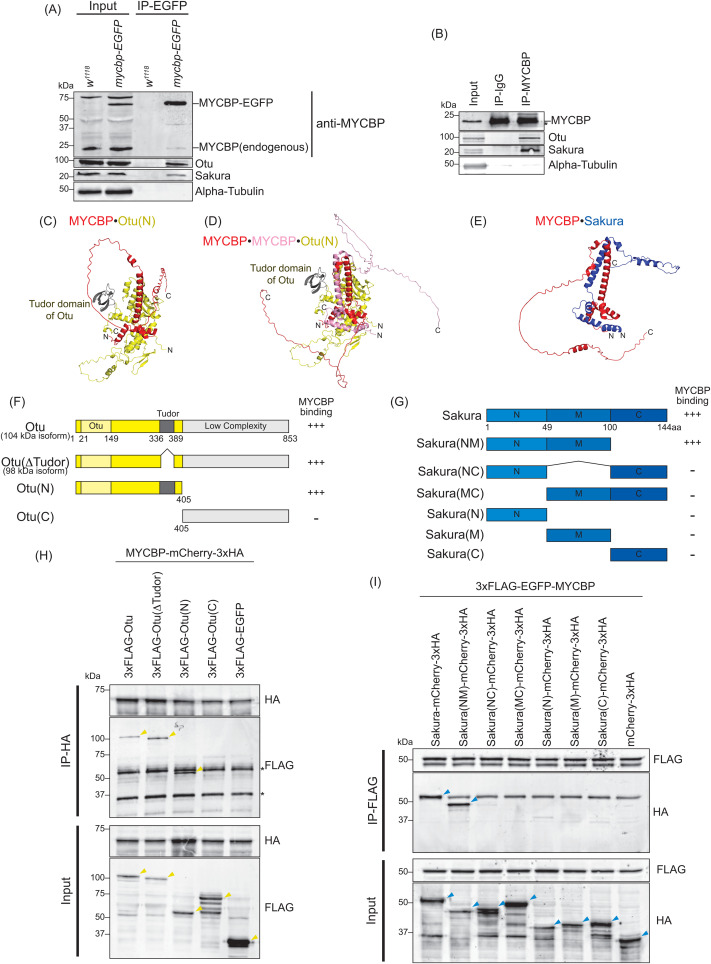
MYCBP interacts with Otu and Sakura. **(A)** Co-immunoprecipitation (co-IP) using anti-GFP magnetic beads followed by Western blotting. Ovary lysates expressing MYCBP-EGFP in *mycbp*^*+/+*^ background and those from *w*^*1118*^ negative control were analyzed. **(B)** Co-IP using anti-MYCBP antibodies and ovary lysates from *w*^*1118*^. IgG was used as a negative control IP. * indicates non-specific bands. **(C-E)** Structures of **(C)** MYCBP•Otu(N), **(D)** MYCBP•MYCBP•Otu(N), and **(E)** MYCBP•Sakura predicted using Alphafold. **(F, G)** Schematic diagrams of full-length and fragment constructs of **(F)** Otu and **(G)** Sakura used in co-IP assays. The binding assay results from (H) is summarized. **(G)** Full-length Sakura and Sakura fragments tested in co-immunoprecipitation assays. N: N-terminal, M: middle, C: C-terminal. The co-IP assay results from (H) and (I) are summarized. **(H)** Co-IP using anti-HA beads followed by Western blotting. S2 cell lysates co-expressing MYCBP-mCherry-3xHA and 3xFLAG-Otu (full-length or fragments) were tested. 3xFLAG-EGFPserved as a negative control. * indicates antibody heavy and light chains. Yellow triangles indicate Otu fragments. **(I)** Co-IP using anti-FLAG beads followed by Western blotting. S2 cell lysates co-expressing 3xFLAG-EGFP-MYCBP and Sakura-mCherry-3xHA (full-length or fragments) were tested. mCherry-3xHA served as a negative control. Blue triangles indicate Sakura fragments.

**Fig 3:**
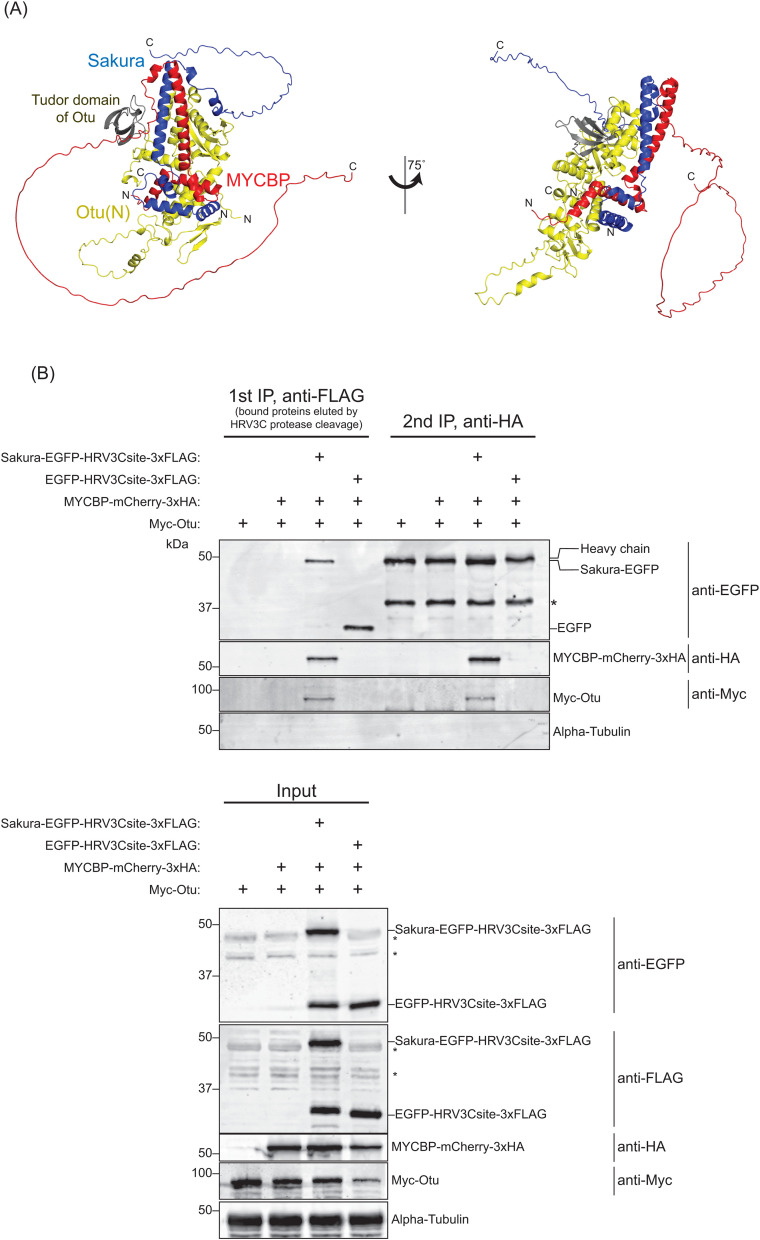
Sequential co-immunoprecipitation shows MYCBP, Sakura, and Otu form a ternary complex. **(A)** MYCBP•Sakura•Otu structure predicted by AlphaFold. **(B)** Sequential co-IP followed by Western blotting. S2 cell lysates co-expressing Sakura-EGFP-HRV3Csite-3xFLAG, MYCBP-mCherry-3xHA, and Myc-Otu were used. EGFP-HRV3csite-3xFLAG served as a negative control. The first IP was performed using anti-FLAG beads and eluted using HRV3C protease. Second IP was performed with anti-HA beads. Non-specific bands are marked with *.

MYCBP was one of the five proteins identified by mass spectrometry following co-immunoprecipitation with Sakura-EGFP from ovary lysates, suggesting a physical interaction between Sakura and MYCBP (Table 1) [8]. We decided to investigate MYCBP since it was predicted to form binary and ternary complexes with Sakura and Otu and structurally resembles Sakura, as we show below (Figs 2C–2E and 3A).

**Table 1:** Number of unique peptide counts detected by mass-spec of Sakura-EGFP co-IP samples.

Identified protein name	Unique peptide counts					
	Sakura-EGFP samples			w1118 samples (negative control)		
	Replicate 1	Replicate 2	Replicate 3	Replicate 1	Replicate 2	Replicate 3
Ovarian tumor (Otu)	44	18	38	0	0	0
CG4679	17	2	8	0	0	0
CG14997	17	2	7	0	0	0
mRpS22	11	2	6	0	1	0
MYCBP	4	2	4	0	1	0

Proteins with at least two unique peptide signals present in all three biological replicates of the Sakura-EGFP samples with no unique peptide signal in any of the three biological replicates of the negative control or only one unique peptide signal in only one of the three biological replicates of the negative control are shown. The same mass-spec data as we presented in our previous publication [8] was used and re-analyzed here.

To examine MYCBP protein expression, we generated polyclonal anti-MYCBP antibodies against the recombinant full-length MYCBP protein. Western blot using this antibody revealed that MYCBP protein is highly expressed in the ovary, including germarium- stage 11 egg chambers, stage 12–13 egg chambers and stage 14 egg chambers (Fig 1C).

### MYCBP is highly expressed in germ cells and cytoplasmic

To analyze MYCBP expression and localization, we created transgenic flies expressing a MYCBP-EGFP fusion protein under the control of the *mycbp* promoter. Oogenesis begins in the germarium, which contains 2–3 GSCs, identifiable by round, unbranched spectrosomes in contact with cap cells (Fig 1A) [5]. In contrast, developing cysts exhibit branched fusomes. We used hu-li tai shao (HTS) antibody to visualize both spectrosomes and fusomes (Fig 1D). Confocal imaging showed that MYCBP*-*EGFP localizes to the cytoplasm of germ cells, including GSCs, cysts, nurse cells, and developing oocytes (Figs 1D, and S1A and S1B). Within egg chambers, MYCBP-EGFP was highly expressed in germline cells (nurse cells and developing oocytes) and was particularly enriched in developing oocytes, whereas its expression was low in was somatic follicle cells and undetectable in somatic stalk cells (S1A and S1B Fig). Consistently, anti-MYCBP staining of control *w*^*1118*^ ovaries revealed similar expression and localization patterns (Figs 1E and S1C).

### MYCBP forms complexes with Otu and Sakura

To identify MYCBP-interacting proteins, we performed co-immunoprecipitation using anti-GFP beads on ovary lysates from MYCBP-EGFP-expressing flies, followed by mass spectrometry. Ovary lysates from *w*^*1118*^ flies served as negative controls. Mass spectrometry identified Otu as the top interactor and also detected Sakura (Table 2).

**Table 2:** Abundance of Proteins identified by mass-spec of MYCBP-EGFP co-IP samples.

Identified protein name	Abundance						log2(Fold-Change)	Adjusted p-value
	MYCBP-EGFP samples			w1118 samples (Negative controls)				
	Replicate 1	Replicate 2	Replicate 3	Replicate 1	Replicate 2	Replicate 3		
MYCBP	29.93	29.53	30.54	16.69	nd	21.67	10.82	1.4E-11
Otu	26.16	25.28	25.68	nd	18.57	20.74	6.05	0.009
Sakura	19.25	18.68	17.27	nd	nd	nd	NA	NA
Sec71	26.94	26.28	27.22	nd	nd	nd	NA	NA
CG15715	24.42	22.41	22.58	nd	nd	nd	NA	NA
Rg	22.61	22.31	23.16	nd	nd	nd	NA	NA
mRpS2	21.30	19.51	20.08	nd	nd	nd	NA	NA
CG7878	20.33	17.92	17.86	nd	nd	nd	NA	NA
Elav	19.98	18.26	19.10	nd	nd	nd	NA	NA
EIF4G2	19.86	18.45	18.94	nd	nd	nd	NA	NA
Rbp9	19.60	18.16	18.86	nd	nd	nd	NA	NA
Whd	19.58	17.81	18.50	nd	nd	nd	NA	NA
Cht10	19.45	18.36	18.85	nd	nd	nd	NA	NA
CG31717	18.82	17.39	18.39	nd	nd	nd	NA	NA
RhoGAP15B	18.72	18.40	19.29	nd	nd	nd	NA	NA
Spg7	17.98	16.94	17.35	nd	nd	nd	NA	NA
Polr3C	17.79	23.48	21.61	nd	nd	nd	NA	NA
Adck1	16.38	14.35	15.44	nd	nd	nd	NA	NA
CG4332	15.74	13.69	13.82	nd	nd	nd	NA	NA

Proteins with adjusted p-value <0.05 or those detected in all MCYBP-EGFP replicates but not in any w1118 replicated are shown.

Western blotting confirmed that both Otu and Sakura co-immunoprecipitate with MYCBP-EGFP in ovary lysates (Fig 2A). Additionally, endogenous MYCBP co-immunoprecipitated with endogenous Otu and Sakura from wild-type ovary lysates (Fig 2B), confirming these interactions in vivo.

Endogenous MYCBP also co-immunoprecipitated with MYCBP-EGFP in ovary lysates, suggesting an interaction between MYCBP proteins (Fig 2A). N-terminally 3xFLAG-EGFP-tagged MYCBP co-immunoprecipitated with C-terminally mCherry-3xHA tagged MYCBP in S2 cells (S2A Fig), confirming the interaction between two MYCBP proteins.

We predicted the structures of the MYCBP•MYCBP, MYCBP•Otu, MYCBP•MYCBP•Otu, and MYCBP•Sakura complexes using AlphaFold [20; 21], which gave us reasonable predicted structures with sufficient inter-molecular interaction without steric hindrance when using the N-terminal region (1–405aa. Otu(N)), but not the C-terminal region, of Otu (Figs 2C–2E, and S3). In the predicted MYCBP•MYCBP structure, two MYCBP molecule have pseudo-symmetric interaction involving mostly their alpha-helices (S3A Fig). In the predicted structures of both MYCBP•Otu and MYCBP•MYCBP•Otu complexes, Tudor domain of Otu is not involved in the direct interaction with MYCBP. To test these, we created epitope-tagged full-length and truncated Otu—Otu(∆Tudor, corresponding to the endogenous 98 kDa isoform), Otu(N), and Otu(C)—and co-expressed them with epitope-tagged full-length MYCBP in S2 cells. (Fig 2F). Co-immunoprecipitation followed by Western blotting showed that all fragments except Otu(C) interacted with MYCBP (Fig 2H), demonstrating the formation of the MYCBP•Otu and/or MYCBP•MYCBP•Otu complex consistent with the AlphaFold prediction.

AlphaFold predicted a pseudo-symmetric alpha-helical interaction between MYCBP and Sakura, involving the N-terminal and middle regions of Sakura (1–100 aa) (Fig 2E). We generated epitope-tagged full-length and truncated Sakura—Sakura(NM), Sakura(NC), Sakura(MC), Sakura(N), Sakura(M), and Sakura(C) —and tested their ability to bind epitope-tagged full-length MYCBP in S2 cells (Fig 2G). Only full-length Sakura and Sakura(NM) interacted with MYCBP (Fig 2I), supporting the predicted MYCBP•Sakura interaction.

AlphaFold also predicted that MYCBP, Sakura, and Otu could form a ternary complex, MYCBP•Sakura•Otu (Fig 3A). To test this, we performed sequential co-immunoprecipitation from S2 cells co-expressing Sakura-EGFP-HRV3Csite-3xFLAG, MYCBP-mCherry-3xHA, and Myc-Otu (Fig 3B). After FLAG-IP and elution by HRV3C protease cleavage, a second HA-IP detected all three proteins, but not when EGFP-HRV3Csite-3xFLAG was used as a control. Additional co-immunoprecipitation showed that MYCBP-mCherry-3xHA specifically bound Myc-Otu, but not Myc-tagged CycA, Bam, EGFP, or *Drosophila* ortholog of MYC (dMyc) (S2B and S2C Fig), confirming the specificity of the interaction. We conclude that MYCBP, Sakura, and Otu form a ternary complex.

Additionally, AlphaFold predicted structures of Sakura•Sakura, Sakura•Otu, and Sakura•Sakura•Otu complexes (S2D–S2F Fig) and we confirmed the interaction between two Sakura proteins using S2 cells (S2D Fig) and that between Sakura and Otu [8].

### Neither MYCBP nor Sakura affects Otu’s deubiquitinase activity in vitro

Otu possesses deubiquitinase activity [18]. While our previous study showed that Sakura does not influence Otu’s deubiquitinase activity in vitro [8], we hypothesized here that MYCBP may affect Otu’s deubiquitinase activity positively or negatively. Using the same Ub-Rhodamine 110-based assay, we tested whether MYCBP, with or without Sakura, affects Otu’s deubiquitinase activity. The addition of recombinant MYCBP, Sakura, or both had no effect on Otu’s deubiquitinase activity, and neither MYCBP nor Sakura showed deubiquitinase activity (S4 Fig). We concluded that neither MYCBP nor Sakura affects Otu’s deubiquitinase activity in vitro.

### *mycbp*^*null*^ mutant flies

**Fig 4:**
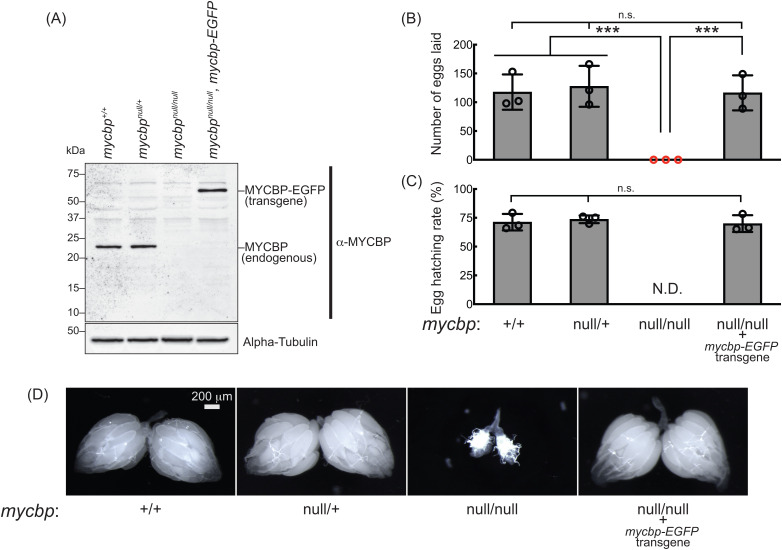
*mycbp*^*null*^ female flies are sterile and have rudimentary ovaries. (A) Western blot of ovary lysates. (B, C) Female fertility assays. (B) The number of eggs laid by test females mated with wild-type (OregonR) males. (C) Hatching rate of the eggs. Mean ± SD (n = 3). P-value < 0.001 (Student’s t-test, unpaired, two-tailed) is indicated by ***. (D) Stereomicroscope images of dissected ovaries. Scale bar: 200 μm.

To examine MYCBP function *in vivo*, we generated a *mycbp* mutant allele (*mycbp*^*null*^) by CRISPR/Cas9-mediated deletion of 38 nucleotides (nts) within the MYCBP coding region, resulting in a frameshift and premature stop codon (Fig 1B). The predicted truncated protein consists of 37 N-terminal amino acids of MYCBP followed by a 47-aa frameshifted segment. This fragment is unlikely to be functional and no stable protein product was detected (Fig 4A), indicating a null mutation. Homozygous *mycbp*^*null/null*^ flies were viable, demonstrating that MYCBP is not essential for survival.

Western blotting confirmed that MYCBP protein is absent in *mycbp*^*null/null*^ ovaries, but present in wild-type (*mycbp*^*+/+*^) and heterozygous controls (*mycbp*^*null/+*^) (Fig 4A). No smaller fragments corresponding to truncated MYCBP were detected in either *mycbp*^*null/+*^ or *mycbp*^*null/null*^, suggesting that the fragment is unstable or not expressed. We performed mosaic analysis using the FLP-FRT system driven by heat shock-inducible FLP (hs-HLP) to generate control (*FRT82B* only) and *mycbp*^*null*^ (*FRT82B, mycbp*^*null*^) germline clones, marked by the absence of GFP, and stained ovaries with anti-MYCBP antibody (S1D Fig). In the same ovarioles of *FRT82B, mycbp*^*null*^ flies, GFP-negative *mycbp*^*null*^ clone cells showed strongly reduced MYCBP staining compared with neighboring GFP-positive control cells; similarly, control clones in *FRT82B*-only flies displayed normal staining (S1D Fig). These results validate both the *mycbp*^*null*^ allele and the anti-MYCBP antibody.

### MYCBP is essential for female fertility

Given MYCBP’s high expression in ovaries (Fig 1C) and its physical interaction with Otu and Sakura (Figs 2 and 3), we hypothesized a role in oogenesis. *mycbp*^*null/null*^ females laid no eggs (Fig 4B and 4C) and their ovaries appeared rudimentary (Fig 4D). Expression of transgenic MYCBP-EGFP in the *mycbp*^*null/null*^ background (*mycbp*^*null/null*^; *mycbp-EGFP*) fully rescued both fertility and ovary morphology (Fig 4B–4D). Western blotting confirmed that these rescue flies expressed MYCBP-EGFP at levels comparable to endogenous MYCBP in control flies (Fig 4A).

MYCBP-EGFP expression in the background of *mycbp*^+/+^ was also detected in testes, including germline cells such as GSCs, where it displayed both broad cytoplasmic distribution and punctate cytoplasmic signals in spermatocytes, but was absent from hub cells (S5A Fig). Similarly, endogenous MYCBP expression in a control *w*^*1118*^ testes showed the same patterm, including punctate localization (S5B Fig). However, *mycbp*^*null/null*^ males were fully fertile (S5C Fig). Therefore, the functional significance of MYCBP expression and localization in testes remains unclear. We conclude that MYCBP is essential for female fertility and normal ovary morphology but is not required for male fertility.

### *mycbp*^*null/null*^ ovaries are germless or tumorous

**Fig 5:**
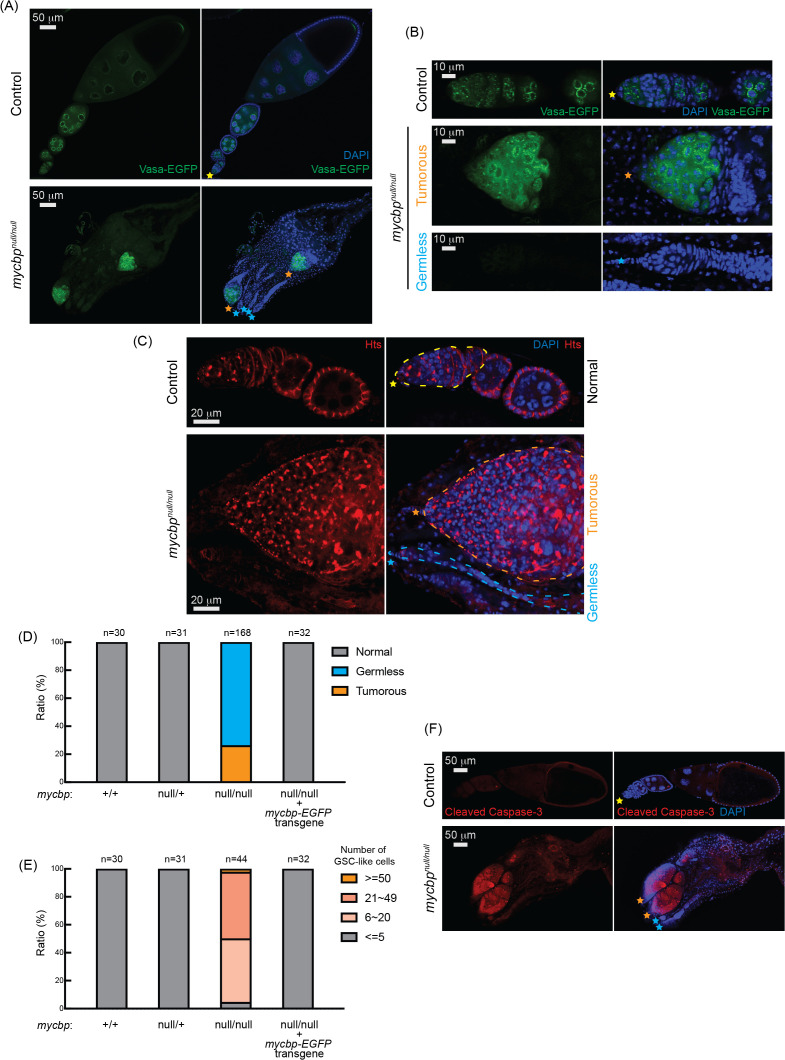
*mycbp*^*null*^ ovaries are germless and tumorous. (A, B) Confocal images of ovaries from control (*mycbp*^*null/+*^) and *mycbp*^*null/null*^ flies expressing Vasa-EGFP. Vasa-EGFP (green) and DAPI (blue). Yellow, orange, and. cyan stars mark normal ovarioles, tumorous, and germless ovarioles, respectively, in Fig 5. (B) Higher-magnification images of germaria. Scale bars: 50 μm (A), 10 μm (B). (C) Confocal images of ovaries from control (*mycbp*^*null/+*^) and *mycbp*^*null/null*^ flies stained with anti-Hts to label spectrosomes and fusomes. Hts (red) and DAPI (blue). Germaria are outlined. Scale bars: 20 μm. (D) Percentage of normal, germless, and tumorous ovarioles in indicated genotypes (ages 2–5 days). (E) Quantification of GSC-like cells per germarium (ages 2–5 days). (F) Confocal images of ovaries stained with anti-cleaved Caspase-3. Cleaved caspase-3 (red) and DAPI (blue). Scale bars: 50 μm.

To investigate the ovary phenotype, we analyzed *mycbp*^*null/null*^ flies expressing a Vasa-EGFP reporter, as Vasa is a known germ cell marker. Some *mycbp*^*null/null*^ ovarioles lacked germ cells (“germless”, cyan stars), while others contained germ cells (Fig 5A and 5B). HTS staining revealed overproliferation of GSC-like cells with round spectrosomes in *mycbp*^*null/null*^ ovarioles containing germ cells (Fig 5C, orange stars), indicative of a “tumorous” phenotype as previously described for mutants of *bam*, *otu*, *sxl*, and *sakura* [9–12,22–26]. Additionally, we observed an excess number of cyst cells with branched fusomes that persisted throughout the ovarioles, suggesting abnormal cyst cell development.

Within the same ovary, both germless and tumorous ovarioles were observed (Fig 5A–5C). 26% of ovarioles were tumorous, while 74% were germless in 2–5-day-old *mycbp*^*null/null*^ flies (n = 168) while all ovarioles in control (*mycbp*^*+/+*^ and *mycbp*^*null/+*^) and *mycbp-EGFP* rescue flies were normal (Fig 5D). The mean number of GSCs or GSC-like cells in 2–5-day-old *mycbp*^*+/+*^, *mycbp*^*null/+*^, and *mycbp-EGFP* rescue was 2.0 ± 0.6, 2.2 ± 0.6, and 2.1 ± 0.5, respectively while that for *mycbp*^*null/null*^ was 21.7 ± 12.7 (p-value <0.001) (S6 Fig). 95% of *mycbp*^*null/null*^ ovarioles containing germ cells had more than five GSC-like cells (Fig 5E). Tumorous ovarioles displayed markedly elevated cleaved Caspase-3 staining, indicative of apoptosis (Fig 5F). In addition, germaria from tumorous *mycbp*^*null/null*^ ovarioles showed increased levels of the mitotic marker Ser10-phosphorylated Histone H3 (pH3), indicating enhanced mitotic cell proliferation (S7A Fig). A similar increase in pH3 signal was observed in germaria of tumorous *sakura*^*null/null*^ ovarioles (S7B Fig). Together, these findings support the idea that MYCBP is required to regulate GSC survival, proliferation, and differentiation.

### Loss of *mycbp* results in loss of piRNA-mediated transposon silencing

piRNAs are produced in germline cells and somatic follicle cells and transcriptionally and post-transcriptionally silence transposon expression through sequence-complementarity [27,28]. Defects in the piRNA pathway result in oogenesis arrest, germ cell loss, rudimentary ovaries, sterility, upregulation (desilencing) of transposons, increased DNA damage, and apoptotic cell death.

In control ovaries, Vasa-EGFP localized to the perinuclear nuage (Fig 5A and 5B), which is essential for piwi-interacting RNA (piRNA) biogenesis. This localization persisted in *mycbp*^*null/null*^ tumorous ovarioles, suggesting that MYCBP is not required for Vasa localization.

**Fig 6:**
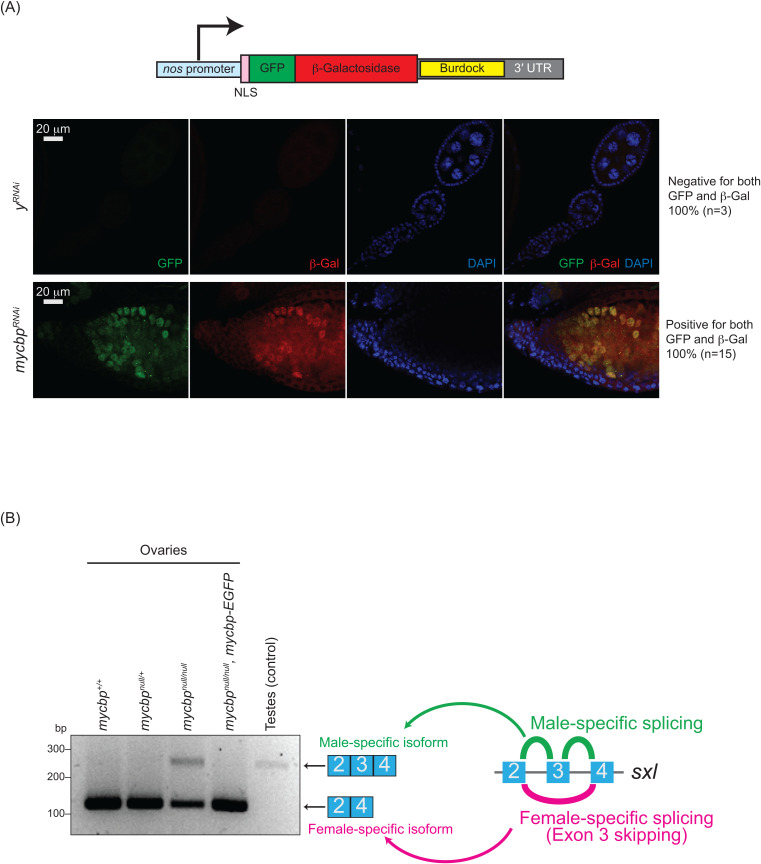
Loss of *mycbp* disrupts piRNA-mediated transposon silencing and *sxl* splicing. **(A)** Schematic of the *Burdock* sensor construct and representative images of sensor expression in ovaries from control (*y*^*RNAi*^) and *mycbp*^*RNAi*^ flies. The *Burdock* sensor harbors a *nanos* promoter, a nuclear localization signal (NLS) appended to GFP and β-gal coding sequences, and a target sequence for *Burdock* piRNAs in the 3’UTR. RNAi was driven in the germline using *UAS-Dcr2*, *NGT-Gal4*, and *nos-Gal4-VP16*. GFP (green), β-gal (red), and DAPI (blue). Scale bars: 20 μm. 0/3 control samples showed sensor activation; 15/15 *mycbp*^*RNAi*^ samples did. **(B)** RT-PCR analysis of *sxl* alternative splicing in ovaries and testes.

To assess MYCBP’s role in the piRNA pathway, we used the *Burdock* transposon sensor, which expresses nuclear GFP and β-galactosidase (β-gal) in germ cells but is silenced by piRNAs (Fig 6A) [29]. In germline-specific *mycbp* RNAi flies (*UAS-Dcr2*, *NGT-Gal4*, *nos-Gal4-VP16 *>* mycbp*^*RNAi*^), we observed strong reporter expression, showing that loss of *mycbp* results in loss of piRNA-mediated transposon silencing.

### Sex-specific *sxl* mRNA alternative splicing is dysregulated in *mycbp*^*null/null*^ ovaries

Sxl is a master regulator of sex determination in *Drosophila* [30–32]. Sex-specific alternative splicing of *sxl* transcripts produces distinct mRNA isoforms: the female-specific, which encodes functional Sxl protein, and the male-specific mRNA isoforms, which does not. In the female germline, loss of Sxl function or disruption of female-specific *sxl* splicing leads to developmental defects, including germline tumors and sterility. Notably, several mutants with tumorous ovariole phenotypes—such as *otu* and *sakura* mutants—exhibit aberrant *sxl* mRNA splicing, resulting in male-specific isoform expression in ovaries and defects in germ cell sexual identity [8,23,14].

As expected, ovaries from control and *mycbp-EGFP* rescue flies expressed only the female-specific *sxl* mRNA isoform, while control testes expressed only the male-specific isoform (Fig 6B). In contrast, *mycbp*^*null/null*^ ovaries showed aberrant expression of the male-specific isoform and a reduced level of the female-specific isoform. These results indicate that female-specific *sxl* splicing is disrupted in the absence of *mycbp*.

### MYCBP is required intrinsically for GSC maintenance and differentiation

**Fig 7:**
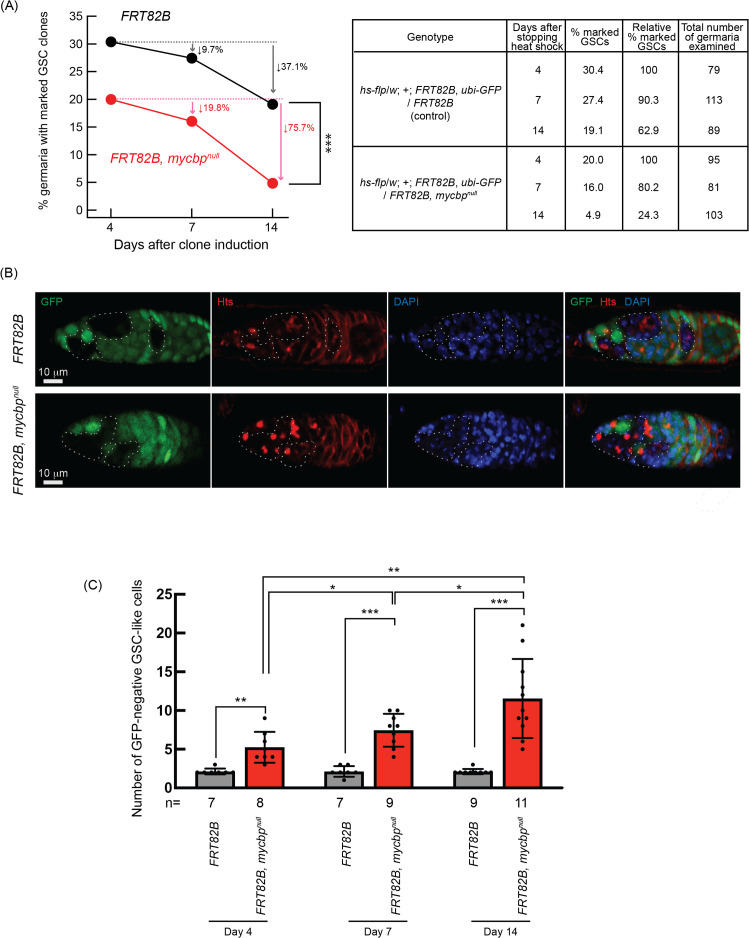
Germline clonal analysis of *mycbp*^*null*^. **(A)** Percentage of germaria with marked (GFP-negative) GSC clones at 4, 7, and 14 days after clone induction. P-value < 0.001 (Chi-squared test) is indicated by ***. **(B)** Confocal images of control and *mycbp*^*null*^ clones. GFP (green), Hts (red), and DAPI (blue). Scale bar: 10 μm. **(C)** Number of marked (GFP-negative) GSC-like cells per germarium containing marked GSCs at 4, 7, and 14 days after clone induction. GSC-like cells containing round spectrosome were identified by anti-Hts staining. P-value < 0.05, < 0.01, and <0.001 indicated by *, **, and *** (Student’s t-test, unpaired, two-tailed).

To test whether *mycbp* functions autonomously in the germline, we performed mosaic analysis using the FLP-FRT system driven by heat shock-inducible FLP (hs-HLP) [15,33)]. GSC maintenance was assessed by generating *mycbp*^*null*^ GSC clones and measuring clone loss over time [8,34,35]. At four days post-induction, 30.4% of GSCs were marked (GFP-negative) in controls (*FRT82B*) and 20.0% were marked in *mycbp*^*null*^ (*FRT82B*, *mycbp*^*null*^), establishing initial clone frequencies (Fig 7A). By day 14, marked controle GSCs declined modestly to 19.1% (37.1% loss), whereas marked *mycbp*^*null*^ GSC clones dropped sharply to 4.9% (75.7% loss), indicating that *mycbp* is intrinsically required for GSC maintenance.

To determine whether *mycbp* is also intrinsically required for GSC differentiation, we quantified the numbers of marked and unmarked GSC-like cells—germline cells with a round spectrosome—in germaria containing marked GSC clones (Fig 7B). Marked *mycbp*^*null*^ GSC-like cells were significantly more abundant than marked controls at all time points (Fig 7C), and their number increased over time, whereas the number of marked control cells did not (Fig 7C). In contrast, the number of unmarked GSC-like cells in germaria containing marked *mycbp*^*null*^ GSC clones did not differ significantly compared with that in germaria containing marked control GSC clones, and their number did not increase over time (S8 Fig). Thus, in the absence of *mycbp*, germline cells exhibit uncontrolled proliferation and tumorous phenotypes. These findings demonstrate that *mycbp* is intrinsically required for both GSCs maintenance and proper differentiation.

### Loss of *mycbp* inhibits Dpp/BMP signaling

**Fig 8:**
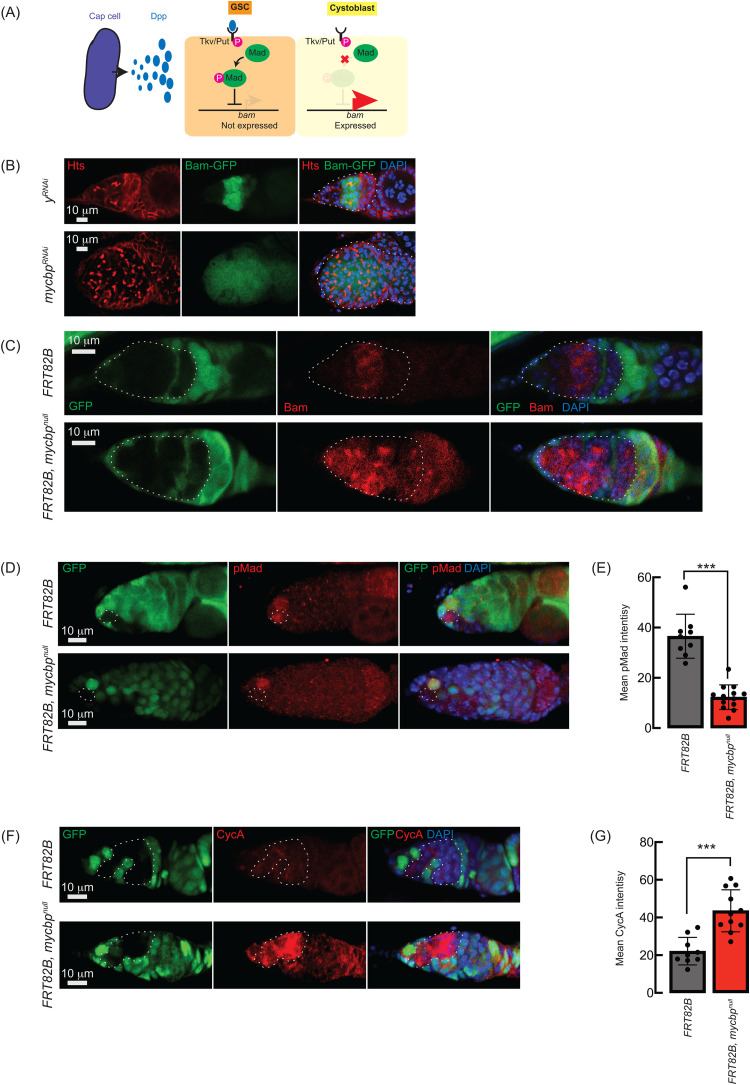
Loss of *sakura* inhibits Dpp/BMP signaling. **(A)** Schematic of Dpp-mediated *bam* repression via pMad activation. Cap cells secrete diffusible Decapentaplegic (Dpp), which is received by its receptor, a heterodimer of Thick vein (Tkv) and Punt (Put), in GSCs. The activated Dpp signaling eventually phosphorylates Mother-against-dpp (Mad). The phosphorylated Mad (pMad) represses the transcription of *bam*. The repression of *bam* in GSCs is crucial for maintaining their stemness. Cystoblasts do not receive Dpp, and Bam expression is crucial for promoting cystoblast differentiation from GSCs. This is the same figure as we used in our previous publication [8]. **(B)** Confocal images of *bam-GFP* reporter expression in control (*y*^*RNAi*^) and *mycbp*^*RNAi*^ ovaries [8]. RNAi was driven in the female germline using *UAS-Dcr2* and *NGT-Gal4*. Bam-GFP (green), Hts (red), and DAPI (blue). Germaria are outlined. Scale bar: 10 μm. **(C, D)** Confocal images of germaria with control and *mycbp*^*null*^ germline clones stained with (C) anti-Bam and (D) anti-pMad. GFP (green), Bam/pMad (red), and DAPI (blue). Scale bar: 10 μm. **(E)** Quantification of pMad intensity in GSC clones. Mean ± SD (n = 9 and 13 for *FRT82B* and *FRT82B, mycbp*^*null*^, respectively). P-value < 0.001 (Student’s t-test, unpaired, two-tailed) is indicated by ***. **(F)** Confocal images of germaria with control and *mycbp*^*null*^ germline clones stained with anti-CycA. GFP (green), CycA (red), and DAPI (blue). Scale bar: 10 μm. **(G)** Quantification of CycA intensity in the germline clones. Mean ± SD (n = 9 and 11 for *FRT82B* and *FRT82B, mycbp*^*null*^, respectively). P-value < 0.01 (Student’s t-test, unpaired, two-tailed) is indicated by **.Clones in C, D, and F are GFP-negative.

The Dpp/BMP signaling pathway plays a central role in regulating GSC self-renewal and differentiation by repressing *bam* in GSCs, which is de-repressed in daughter cystoblasts (Fig 8A) [5,36]. We hypothesized that the germless and tumorous phenotypes observed in *mycbp*^*null*^ ovaries might result from misregulation of this pathway. To test this, we RNAi-knocked down *mycbp* in the germline using *UAS-Dcr2* and *NGT-Gal4* in the flies carrying the *bam-GFP* reporter [37]. In controls (*y*^*RNAi*^), Bam-GFP expression was restricted to 8-cell cysts and absent in later stages (Fig 8B). In contrast, *mycbp* knockdown ovaries exhibited persistent Bam-GFP expression throughout the germarium, including in GSCs (Fig 8B).

We confirmed this using the FLP-FRT-mediated *mycbp*^*null*^ clones and performed anti-Bam immunostaining. In control clones (*FRT82B*), Bam expression was confined to 8-cell cysts, whereas in *mycbp*^*null*^ clones (*FRT82B*, *mycbp*^*null*^), Bam was aberrantly expressed throughout the germarium, including in GSCs (Fig 8C). This suggests that in the absence of *mycbp*, Bam expression is no longer repressed by Dpp/BMP signaling in GSCs, resulting in GSC loss.

Dpp/BMP signaling represses *bam* transcription via the transcription factor Mad, which, when phosphorylated (pMad), translocates into the nucleus to repress *bam* transcription [5,38,39]. anti-pMad staining revealed significantly reduced pMad in *mycbp*^*null*^ GSC clones compared to control GSCs (Fig 8D and 8E), indicating compromised BMP signaling and transcriptional *bam* de-respression, though Bam is also known to be regulated post-transcriptionally [40].

Previous studies have shown that ectopic expression of a stable form of CycA leads to germ cell loss [41]. This germ cell loss phenotype is also observed upon ectopic *bam* expression in GSCs [34,42,43]. It was reported that Bam associates with Otu to promote deubiquitination and stabilization of CycA [18]. Anti-CycA staining showed significantly elevated CycA in *mycbp*^*null*^ clones relative to neighboring wild-type cells in the same germarium (*FRT82B*, *mycbp*^*null*^) and control clones in control germarium (*FRT82B*) (Fig 8F and 8G), suggesting that Bam misexpression in *mycbp*^*null*^ stabilizes CycA.

### *mycbp* is required for oogenesis beyond GSCs and germline cysts

**Fig 9:**
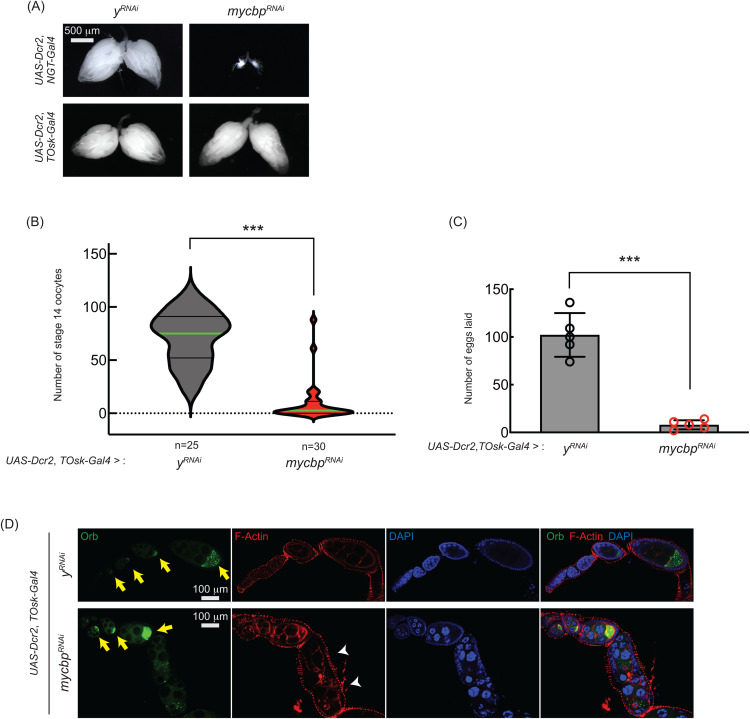
*mycbp* is important for late oogenesis. (A) Stereomicroscope images of dissected ovaries. Scale bar: 500 μm. *y*^*RNAi*^ served as a control. (B, C) Number of stage 14 oocytes per fly (B) and eggs laid (C) in *mycbp* RNAi knockdown usingby *UAS-Dcr2* and *TOsk-Gal4*. Mean ± SD (n = 5). P-value < 0.001 (Student’s t-test, unpaired, two-tailed) is indicated by ***. (D) Confocal images of ovaries stained with phalloidin (F-Actin, red), anti-Orb (Green), and DAPI (blue). Yellow arrows indicate normal Orb localization; white arrowheads indicate cytoskeletal disorganization and loss of Orb localization. Scale bar: 100 μm.

Both *mycbp*^*null/null*^ mutation and *mycbp* germline RNAi knockdown using *UAS-Dcr-2* and *NGT-Gal4*, which initiates RNAi in germline cells starting from GSCs, resulted in rudimentary ovaries lacking late-stage germline cells (Figs 4D and 9A), preventing assessment of MYCBP’s role beyond the germarium. However, because MYCBP is expressed throughout egg chamber development, (Figs 1C–1E and S1), we hypothesized it may also function in later germline stages.

To test this, we used *TOsk-Gal4* (a combination of *osk-Gal4* and *αTub67C-Gal4*) to RNAi-knockdown *mycbp* in germline cells starting from germarium region 2b onward, thereby sparing GSCs and early cysts [44]. While *TOsk-Gal4*-driven *mycbp* RNAi did not affect ovary morphology (Fig 9A), it dramatically reduced stage 14 oocyte production and egg laying compared to control RNAi (*y*^*RNAi*^) (Fig 9B and 9C), suggesting that *mycbp* is important for oogenesis beyond the germline cyst stage.

**Fig 10:**
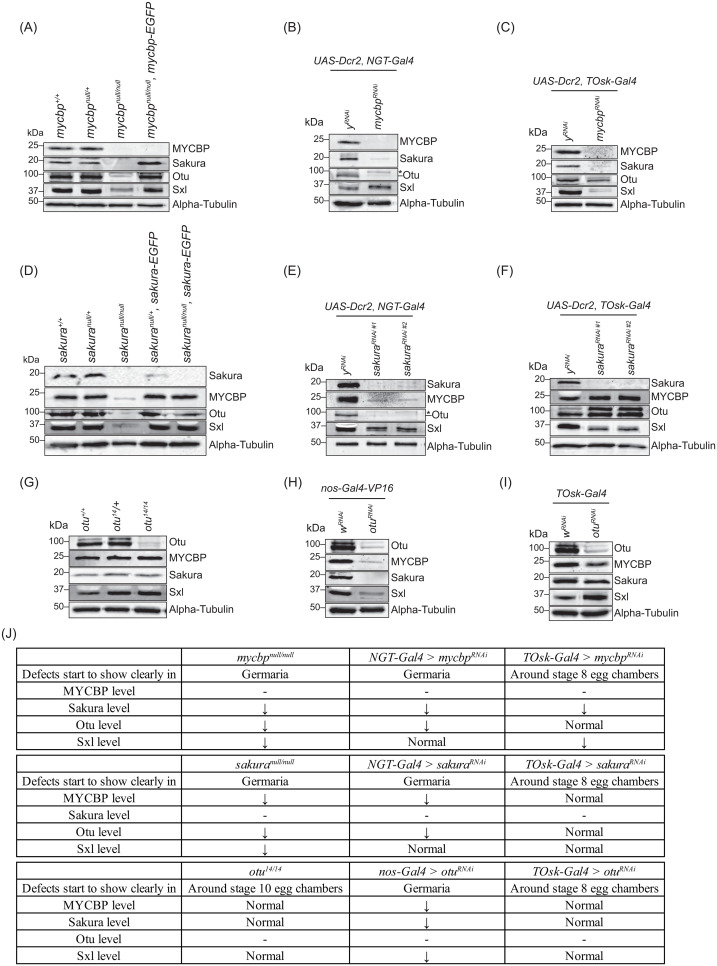
MYCBP is required for Sakura level. Western blot of ovary lysates. The *sakura RNAi* #1 (VDRC: v39727) and #2 (VDRC: v103660). Non-specific bands are marked (*). Sakura and alpha-Tubulin images in **(E)**, Sakura, Otu, and alpha-Tubulin images in **(F)**, Otu and alpha-Tubulin images in **(H)**, Sakura, Otu, and alpha-Tubulin images in **(I)**, are from [8]. Results are summarized in **(J)**.

We also analyzed Oo18 RNA-binding protein (Orb), a marker of oocyte identity. In controls, Orb localized to the posterior within stage ~4–8 egg chambers (Fig 9D, yellow arrows). In *TOsk-Gal4 *> *mycbp*^*RNAi*^ ovaries, Orb localization appeared normal through stage ~6 (Fig 9D, yellow arrows) but was lost by stage ~8 with signs of cytoskeletal disorganization (Fig 9D, white arrowheads). In addition, nurse cell nuclei appeared abnormal in *TOsk-Gal4 *> *mycbp*^*RNAi*^ ovaries (Fig 9D), suggesting apoptosis, similar to what we observed in *mycbp*^*null*^ mutant ovaries (Fig 5F). These defects likely contribute to the impaired oogenesis observed (Fig 9B and 9C). RNAi efficiency was confirmed by Western blots (Fig 10B and 10C).

Similarly, in *TOsk-Gal4 *> *otu*^*RNAi*^ ovaries [8], Orb localization and cytoskeletal organization were normal up to approximately stage 6 but became dysregulated by stage ~8 (S9 Fig). We conclude that MYCBP plays essential roles in oogenesis beyond early germline stages, similar to Sakura and Otu [8].

### MYCBP is crucial for Sakura levels

Given the formation of the protein complexes among MYCBP, Sakura, and Otu, as well as the shared phenotypes observed in their respective mutants, we investigated whether their protein levels are interdependent. We also assessed Sxl protein levels due to the dysregulation of *sxl* splicing ovserved in their mutants. We performed Western blots using ovary lysates from genetic mutants (*mycbp*^*null/null*^, *sakura*^*null/null*^, and *otu*^*14/14*^) and from RNAi-mediated knockdowns driven by germline-specific Gal4 drivers. Specifically, we used *NGT-Gal4* and *nos-Gal4-VP16* to deplete the proteins starting from GSCs, and *TOsk-Gal4* to deplete proteins from germarium region 2b onward (Fig 10). *otu*^*14*^ is a ”differentiated (DIF)” class of allele with defects in egg chamber maturation [14,17,45,46)].

In *mycbp*^*null/null*^ ovaries, Sakura and Otu levels were severely reduced, and Sxl levels were moderately reduced (Fig 10A). In *NGT-Gal4 > mycbp*^*RNAi*^ ovaries, Sakura and Otu levels were markedly reduce, while Sxl levels remain unchanged (Fig 10B). In *TOsk-Gal4 > mycbp*^*RNAi*^ ovaries, Sakura and Sxl levels were reduced, but Otu was not affected (Fig 10C). There findings indicate that MYCBP is essential for Sakura levels and may also influence Otu and Sxl levels in a developmental stage-dependent manner.

In *sakura*^*null/null*^ ovaries, MYCBP, Otu, and Sxl levels were severely reduced (Fig 10D). In *NGT-Gal4 > sakura*^*RNAi*^ ovaries, MYCBP and Otu were strongly reduced, but Sxl levels remained unaffected (Fig 10E). In *TOsk-Gal4 > sakura*^*RNAi*^ ovaries, Sxl was reduced, while MYCBP and Otu levels were unchanged (Fig 10F). There data suggest that Sakura may support the expression or stability of MYCBP, Otu, and Sxl proteins in a stage-specific manner.

In *otu*^*14/14*^ ovaries, MYCBP, Sakura and Sxl levels were unchanged (Fig 10G). In contrast, in *nos-Gal4-VP16 > otu*^*RNAi*^ ovaries, all three proteins were reduced (Fig 10H), whereas in *TOsk-Gal4 > otu*^*RNAi*^ ovaries, their levels remained unchanged (Fig 10I). There results suggest that Otu may contribute to the expression and/or stability of MYCBP and Sakura proteins under certain developmental conditions. The results are summarized in Fig 10J.

It is important to note that whole ovary lysates include both germline and somatic cells. While Sakura and Otu are expressed exclusively in germ cells in ovaries, MYCBP was enriched in germline cells but was also lowly detectable in somatic follicle cells (Figs 1E, and S1A and S1B) [8]. All Gal4 drivers used (*NGT-Gal4*, *nos-Gal4-VP16* and *TOsk-Gal4*) are germline-specific. Furthermore, ovaries from *mycbp*^*null/null*^, *sakura*^*null/null*^, *NGT-Gal4 > mycbp*^*RNAi*^*, NGT-Gal4 > sakura*^*RNAi*^, and *nos-Gal4-VP16 > otu*^*RNAi*^ flies are significantly smaller and degenerated compared to controls (Fig 4D) [8], complicating the interpretation of Western blot results due to tissue loss or altered cell composition. Therefore, we next pursued alternative approaches to more precisely determine their protein level interdependency.

### MYCBP is required for Sakura level and Sakura is important for MYCBP level and its posterior localization within egg chambers

**Fig 11:**
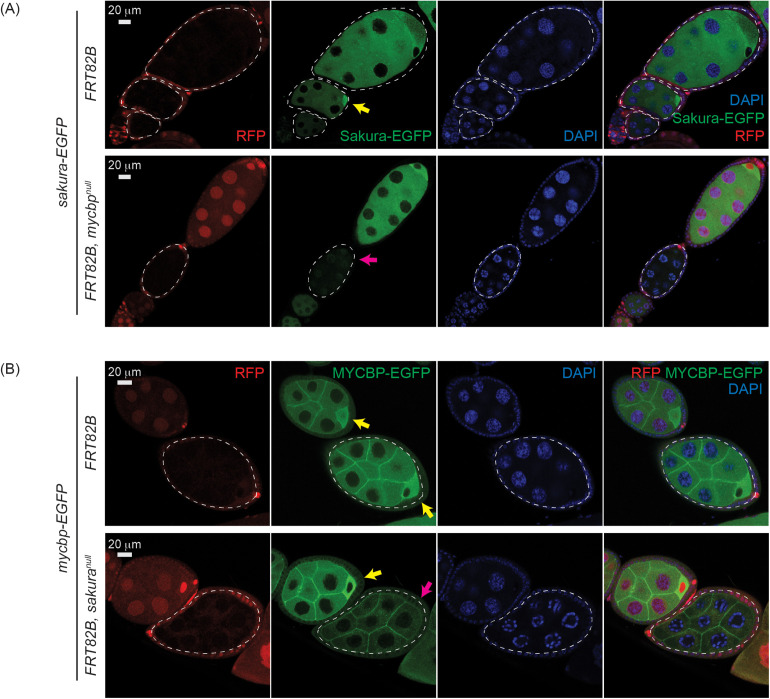
Mutual dependance of MYCBP and Sakura level and localization. **(A)** Confocal images of egg chambers with control and *mycbp*^*null*^ germline clones expressing Sakura-EGFP. Marked clones (RFP-negative) are outlined. RFP (red), Sakura-EGFP (green), and DAPI (blue). Scale bar: 20 μm. Yellow arrow: normal Sakura-EGFP posterior enrichment; magenta arrow: reduced Sakura-EGFP signal. Fly genotypes used: *hs-flp*/*w*; *sakura-EGFP*/ + ; *FRT82B*, *ubi-RFP*/*FRT82B*. *hs-flp*/*w*; *sakura-EGFP*/ + ; *FRT82B*, *ubi-RFP*/*FRT82B*, *mycbp*^*null*^. **(B)** Confocal images of egg chambers with control and *sakura*^*null*^ germline clones expressing MYCBP-EGFP. Marked clones (RFP-negative) are outlined. RFP (red), MYCBP-EGFP (green), and DAPI (blue). Scale bar: 20 μm. Yellow arrow: normal MYCBP-EGFP posterior enrichment; magenta arrow: reduced levels and loss of posterior localization of MYCBP-EGFP. Fly genotypes used: *hs-flp*/*w*; *mycbp-EGFP*/ + ; *FRT82B*, *ubi-RFP*/*FRT82B*. *hs-flp*/*w*; *mycbp-EGFP*/ + ; *FRT82B*, *ubi-RFP*/*FRT82B*, *sakura*^*null*^.

We examined how MYCBP and Sakura influence their protein level and localization within egg chambers each other using FLP-FRT-mediated mosaic analysis. In flies expressing Sakura-EGFP, its signal was markedly reduced in *mycbp*^*null*^ clones (*FRT82B*, *mycbp*^*null*^) compared to control clones (*FRT82B*), confirming MYCBP is essential for Sakura protein level (Fig 11A).

Conversely, in flies expressing MYCBP-EGFP, its level was decreased and its posterior localization was lost in *sakura*^*null*^ clones (*FRT82B*, *sakura*^*null*^) compared in control clones (*FRT82B*) (Fig 11B), indicating that Sakura is required for MYCBP level and its posterior localization within egg chambers.

### MYCBP and Sakura are required for Otu localization to developing oocytes

**Fig 12:**
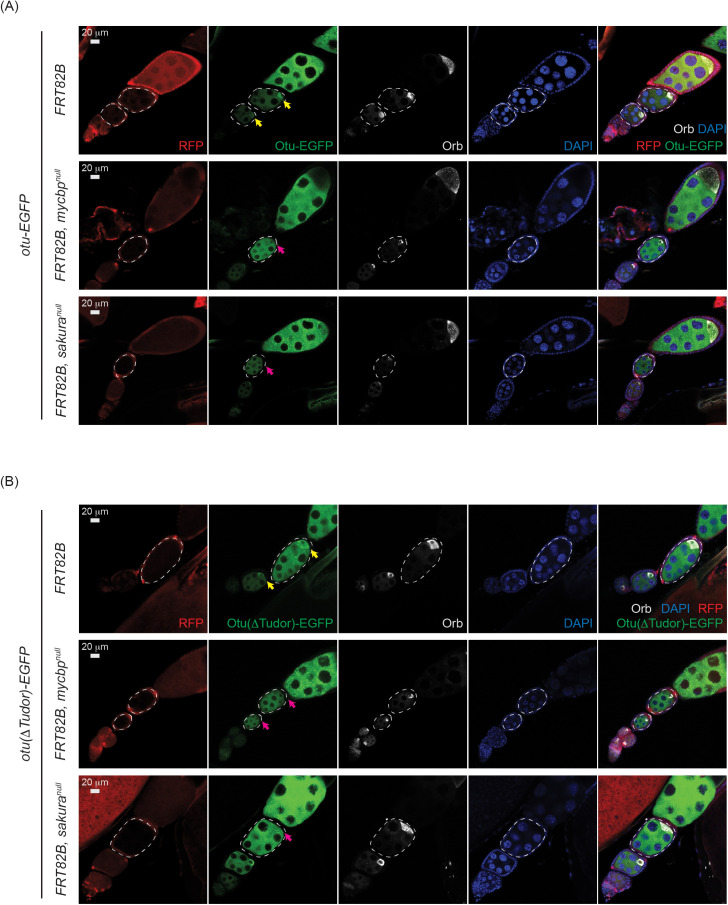
Otu localization to the developing oocyte depends on MYCBP and Sakura. Confocal images of egg chambers with control, *mycbp*^*null*^, and *sakura*^*null*^ germline clones expressing **(A)** Otu-EGFP or **(B)** Otu(ΔTudor)-EGFP. Marked clones (RFP-negative) are outlined. RFP (red), Otu-EGFP or Otu(ΔTudor)-EGFP (green), Orb (white), and DAPI (blue). Scale bar: 20 μm. Yellow arrows: normal posterior enrichment of Otu-EGFP and Otu(ΔTudor)-EGFP; magenta arrows: loss of localization. Orb localization remains intact in mutant clones. Fly genotypes used: *hs-flp*/*w*; *otu-EGFP*/ + ; *FRT82B*, *ubi-RFP*/*FRT82B*. *hs-flp*/*w*; *otu-EGFP*/ + ; *FRT82B*, *ubi-RFP*/*FRT82B*, *mycbp*^*null*^. *hs-flp*/*w*; *otu-EGFP*/ + ; *FRT82B*, *ubi-RFP*/*FRT82B*, *sakura*^*null*^. *hs-flp*/*w*; *otu(*Δ*Tudor)-EGFP*/ + ; *FRT82B*, *ubi-RFP*/*FRT82B*. *hs-flp*/*w*; *otu(*Δ*Tudor)-EGFP*/ + ; *FRT82B*, *ubi-RFP*/*FRT82B*, *mycbp*^*null*^. *hs-flp*/*w*; *otu(*Δ*Tudor)-EGFP*/ + ; *FRT82B*, *ubi-RFP*/*FRT82B*, *sakura*^*null*^.

We examined Otu levels and localization within germaria and egg chambers using *otu-EGFP* and *otu(∆Tudor)-EGFP* transgenes [8] in control, *mycbp*^*null*^, and *sakura*^*null*^ clones. For egg chambers, we additionally stained for Orb, which marks developing oocytes. Otu-EGFP and Otu(∆Tudor)-EGFP levels appeared similar across all genotypes in both the germarium, including in GSCs, and egg chambers (Figs 12 and S10), indicating that MYCBP and Sakura do not affect Otu protein levels. However, Otu-EGFP and Otu(∆Tudor)-EGFP failed to localize to the posterior within the egg chambers in *mycbp*^*null*^ and *sakura*^*null*^ clones (Fig 12). Importantly, Orb remained properly localized in these clones, suggesting that oocyte specification was intact. Thus, MYCBP and Sakura are dispensable for Otu level but are required for its proper localization to developing oocytes.

### MYCBP and Sakura are required for Sxl expression in germline cells, including GSCs

**Fig 13:**
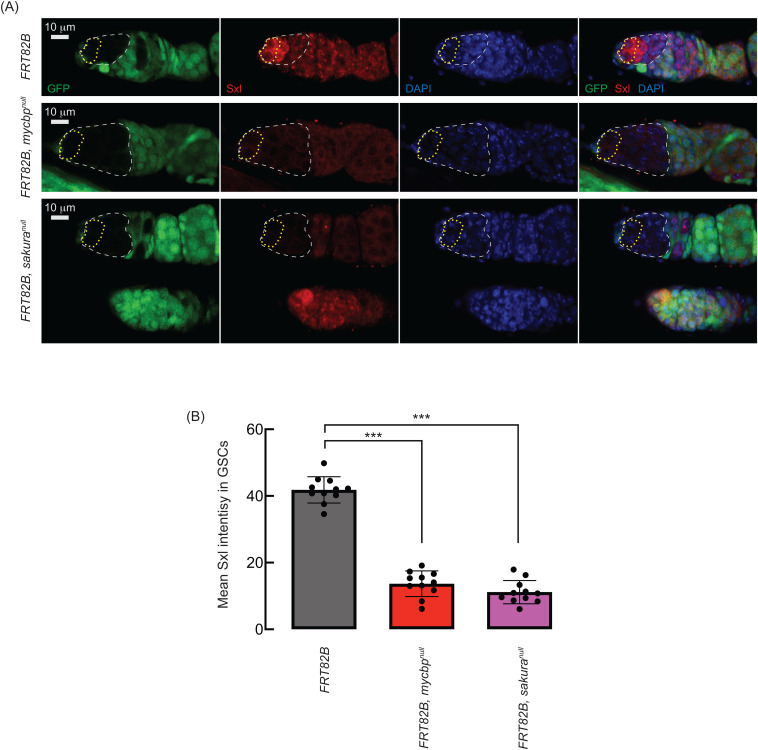
Sxl expression in germaria depends on MYCBP and Sakura. **(A)** Confocal images of germaria with control, *mycbp*^*null*^, and *sakura*^*null*^ germline clones. Marked clones (GFP-negative) are outlined with white and marked GSC clones are outlined with yellow. GFP (green), Sxl (red), and DAPI (blue). Scale bar: 10 μm. Fly genotypes used: *hs-flp*/*w*; + ; *FRT82B*, *ubi-RFP*/*FRT82B*. *hs-flp*/*w*; + ; *FRT82B*, *ubi-RFP*/*FRT82B*, *mycbp*^*null*^. *hs-flp*/*w*; + ; *FRT82B*, *ubi-RFP*/*FRT82B*, *sakura*^*null*^. **(B)** Quantification of Sxl intensity in the GSC clones. Mean ± SD (n = 11). P-value < 0.001 (Student’s t-test, unpaired, two-tailed) is indicated by ***.

We examined Sxl protein levels in germ cells, including GSCs, within the germarium using control, *mycbp*^*null*^, and *sakura*^*null*^ mosaic clones (Fig 13). In marked control clones (*FRT82B*), Sxl was highly expressed in GSCs and cystoblasts, as well as in unmarked GSCs and cystoblasts in control germaria (lower germaria in the *FRT82B, sakura*^*null*^ panels) (Fig 13A). In contrast, Sxl levels were markedly reduced in *mycbp*^*null*^ and *sakura*^*null*^ clone cells, both in GSCs (outlined in yellow) and in differentiating germline cells (outlined in white) (Fig 13A and 13B). These results demonstrate that MYCBP and Sakura are required for proper Sxl expression in germline cells, including in GSCs.

## Discussion

**Fig 14:**
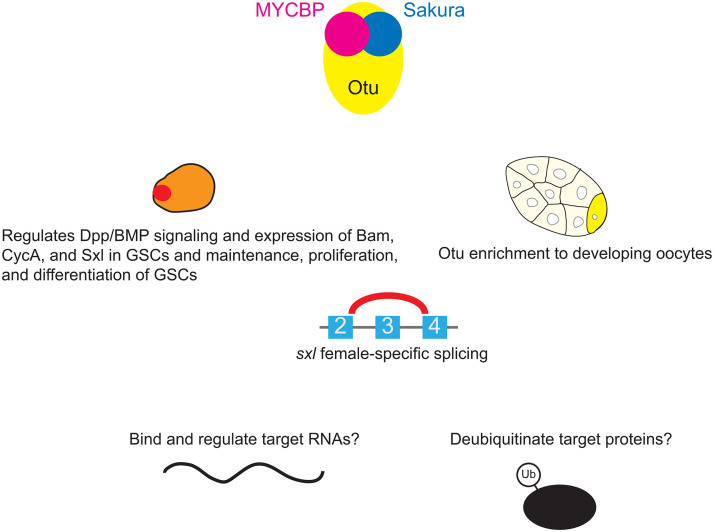
Model of function of MYCBP•Sakura•Otu complex. We propose that MYCBP•Sakura•Otu complex regulates Dpp/BMP signaling and expression of Bam, CycA, and Sxl in GSCs, including playing a crucial role in *sxl* female-specific splicing, and thereby regulates maintenance, proliferation, and differentiation of GSCs and that the MYCBP•Sakura•Otu complex formation is important for enrichment of Otu to developing oocytes in egg chambers, which is crucial for proper oogenesis. MYCBP•Sakura•Otu complex may bind and regulate target RNAs and deubiquitinate target proteins.

We previously showed that Sakura and Otu form a protein complex [8]. In this study, we identified MYCBP, encoded by the previously uncharacterized gene *CG17202*, as a binding partner of both Otu and Sakura. Our data support that MYCBP binds with itself, Sakura, and Otu, forming binary and ternary complexes, including the MYCBP•Sakura•Otu ternary complex. Structural predictions suggest that MYCBP and Sakura resemble each other and engage in a pseudo-symmetric interaction. Mutations in *mycbp*, *otu*, and *sakura* result in strikingly similar phenotypes, and all three proteins are highly expressed in germline cells of the ovary, localize to the cytoplasm, and are enriched in developing oocytes. These observations strongly indicate that MYCBP, Sakura, and Otu function cooperatively in the germline during oogenesis. We propose that the MYCBP•Sakura•Otu complex regulates Dpp/BMP signaling and the expression of Bam, CycA, and Sxl in GSCs, including playing a crucial role in female-specific *sxl* splicing, thereby controlling GSC maintenance, proliferation, and differentiation (Fig 14). Additionally, we propose that formation of the MYCBP•Sakura•Otu complex is important for the enrichment of Otu in developing oocytes within egg chambers, which is essential for proper oogenesis. MYCBP•Sakura•Otu complex may bind and regulate target RNAs and deubiquitinate target proteins.

Since there are multiple protein association states that can be possibly formed among MYCBP, Sakura, and Otu, including MYCBP alone, Sakura alone, Otu alone, MYCBP•MYCBP, Sakura•Sakura, MYCBP•Sakura, MYCBP•Otu, MYCBP•MYCBP•Otu, Sakura•Otu, Sakura•Sakura•Otu, and MYCBP•Sakura•Otu, the relative expression levels among the three proteins and potential regulatory mechanisms for their interaction may determine the relative abundance of these multiple protein complexes, which could be critically important for GSC maintenance and differentiation and oogenesis.

Both tumorous and germless ovarioles were observed in the *mycbp*, *otu*, and *sakura* mutant ovaries (Fig 5) [8]. While upregulation of Bam and CycA (Fig 8) and activation of the apoptotic pathway (Fig 5F) may underlie the germless phenotypes, the mechanism leading to tumorous ovarioles remains unclear. The relative balance of the multiple protein complexes formed by MYCBP, Sakura, and Otu may be differently disrupted among ovarioles within the same mutant, resulting in both tumorous and germless phenotypes. Furthermore, tumorous ovarioles may eventually become germless due to germline apoptosis. The precise mechanism by which these two distinct phenotypes arise within the same mutant ovaries warrants future investigation.

In *mycbp*^*null*^ germline clone cells, Sakura protein levels were severely reduced, and Otu lost its localization to developing oocytes, despite unchanged Otu protein levels and normal posterior localization of Orb (Figs 11A and 12, and S10). These results indicate that MYCBP is required for Sakura protein expression and/or stability, as well as for proper Otu localization. Similarly, in *sakura*^*null*^ germline clones, MYCBP levels were reduced, and both MYCBP and Otu lost their posterior localization, again without affecting Otu levels or Orb localization (Figs 11B and 12, and S10), indicating that Sakura is crucial for MYCBP protein expression and/or stability, as well as for proper MYCBP and Otu localization to developing oocytes. These mutual dependencies of protein expression/stability and oocyte localization among MYCBP, Sakura, and Otu further support the model that they function as protein complexes.

Although MYCBP and Sakura did not directly affect Otu’s deubiquitinase activity in vitro using Ub-Rhodamine 110 as a model substrate (S4 Fig), this does not rule out the possibility that they influence Otu’s enzymatic activity in vivo. For instance, they may modulate Otu’s substrate specificity. Previous work has shown that Otu also interacts with Bam—primarily through its Otu domain—to form a deubiquitinase complex that deubiquitinates and thereby stabilizes CycA, promoting GSC differentiation [18]. It is possible that MYCBP and Sakura regulate the interaction between Otu and Bam and/or modulate the enzymatic activity of the Otu•Bam complex. For example, binding of MYCBP and/or Sukura to Otu may be mutually exclusive with Bam binding. Alternatively, MYCBP, Sakura, Otu, and Bam might form a ternary complex, while we found that MYCBP does not bind Bam (S2B Fig). Further studies are required to elucidate whether and how MYCBP and Sakura influence Otu’s protein interactions and enzymatic function.

Otu also functions as an RNA-binding protein, and its deubiquitinase activity is enhanced by RNA binding [19]. Sxl controls both alternative mRNA splicing and translation of downstream targets, and promotes its own expression via a positive autoregulatory loop [47–50]. We and others have shown that female-specific splicing of *sxl* mRNA is disrupted in *mycbp*, *sakura* and *otu* mutant ovaries, leading to production of the male-specific isoform (Fig 6B) [8,23]. Consistent with these findings, we found that Sxl protein expression in germline cells in germaria including GSCs depends on MYCBP and Sakura (Fig 13). However precise mechanism how MYCBP, Sakura, and Otu play an essential role in Sxl expression remains unknown. Bam, together with Bgcn, Mei-P26, and Sxl, binds *nanos* mRNA—a key stem cell maintenance—and represses its translation after germ cells exit the niche [51–53]. Identifying the RNA targets and deubiquitinase substrates of Otu beyond CycA and how MYCBP and Sakura regulate these Otu’s activities will be critical to understanding their roles in oogenesis and other developmental processes. MYCBP•Sakura•Otu complex may bind directly to RNAs and regulate post-transcriptional processes such as *sxl* alternative splicing and translational control of oogenic RNAs.

Sakura is exclusively expressed in female germline cells including GSCs [8], and MYCBP is also highly expressed in these cells (Fig 1). However, MYCBP is additionally expressed at lower levels in somatic follicle cells in egg chambers and in other tissues, including testes, and Otu is broadly expressed in various tissues such as the gut and testis as well as in female germline cells in ovaries [46]. These differential expression patterns suggest that Otu may have tissue-specific functions depending on the presence or absence of MYCBP and Sakura.

Transposons pose significant threat to genomic stability by inducing DNA damage if not properly silenced [54]. piRNAs suppress transposons through transcriptional and post-transcriptional silencing mechanisms, thus preserving genome integrity [27,28]. Loss of piRNA function results in transposon derepression, increased DNA damage, germ cell apoptosis, arrested oogenesis, and sterility [55,56]. Because damaged germ cells can transmit harmful mutations to the next generation, selective elimination of defective germ cells is critical for maintaining germline integrity of a species [57,58]. We found that loss of function of *mycbp*, *sakura or otu* impairs piRNA-mediated transposon silencing (Fig 6A) and causes apoptosis (Fig 5F) [8]. Thus, the germless phenotypes may arise, at least in part, from activation of a transposon-induced apoptotic elimination program. It will be important to investigate whether MYCBP, Sakura, and Otu’s have any direct roles in the piRNA pathway.

MYCBP and Otu are conserved through human (human MYCBP, also known as AMY-1, and OTUD4) while Sakura is not. Human MYCBP was suggested to bind via its C-termina region to the N-terminal region of C-MYC and stimulate the activation of E-box-dependent transcription by C-MYC. [59]. However, we showed that *Drosophila* MYCBP does not bind *Drosophila* ortholog of MYC (dMyc) (S2C Fig). Large scale protein interaction studies indicated that human MYCBP and OTUD4 associate [60]. Alphafold suggested that human MYCBP and OTUD4 form complexes, MYCBP•OTUD4 and/or MYCBP•MYCBP•OTUD4, via the N-termina region of OTUD4 (S3I and S3J Fig), suggesting the evolutionary conserved interaction between MYCBP ortholog and Otu ortholog.

After we published our studies in which we identified, characterized, and named Sakura, in *bioRxiv* and *eLife* in 2024 (https://www.biorxiv.org/content/10.1101/2024.10.04.616675v2, https://elifesciences.org/reviewed-preprints/103828v1), the Buszczak group reported related studies in 2025, referring to Sakura as Burbon [61]. Consistent with both our previous [8] and current findings, they concluded that Sakura, MYCBP, and Otu form a ternary complex and function together to regulate germline differentiation, including promoting Sxl expression. While our overall conclusions align, there are notable differences as well. Mercer et al. proposed that Sakura stabilizes Otu and facilitates physical interactions between Otu and MYCBP. Their conclusions were based on observations that Otu-GFP levels in the germarium driven by *nos-Gal4 > UASp-otu::GFP* were markedly reduced in *sakura* mutants, and that MYCBP and Otu failed to in S2 cells unless Sakura was co-expressed [61]. While we also observed a reduction of endogenous Otu in whole ovary lysates of *sakura* and *mycbp* null mutants and NGT-Gal4 driven RNAi by Western blot (Fig 10), interpretation of these data is complicated by the severe ovarian degeneration in these mutants. In contrast, our mosaic clone analyses—using Otu-EGFP transgene expressed from the *otu* promoter—clearly demonstrated that Otu protein levels were not reduced in *mycbp*^*null*^ or *sakura*^*null*^ germline cells within germaria or egg chambers, although Otu enrichment in developing oocytes was lost (Figs 12 and S10). Furthermore, we showed that MYCBP and Otu physically associate in S2 cells without Sakura (Fig 2H), unlike the findings reported in Mercer et al. This discrepancy may reflect differences in epitope-tagging strategies. Our results indicate that neither Sakura nor MYCBP is required for Otu stability of for the physical interaction between Otu and MYCBP or Sakura.

In summary, our study identifies and characterizes evolutionary conserved MYCBP as a novel and essential regulator of *Drosophila* oogenesis. Together with Sakura and Otu, MYCBP likely orchestrates germline cell fate decision, maintenance, and differentiation.

## Materials and methods

### Fly strains

We generated the *mycbp*^*null*^ strain by introducing indels into the MYCBP coding region using the CRISPR/Cas9 genome editing, as previously reported [8,62–66]. The transgenic *mycbp*-*EGFP* strain was established following previously published methods [65,67,68]. A DNA fragment containing the MYCBP coding sequence flanked by ~1.2 kb upstream and ~1 kb downstream genomic sequences was cloned. EGFP was fused in-frame to the C-terminus of the MYCBP coding sequence. The construct was inserted into a pattB plasmid and integrated at the 25C6 landing site using the attP40 fly line and the PhiC31 integrase system (BestGene).

Transgenic *otu-EGFP, otu(∆Tudor)-EGFP,* and *sakura-EGFP* strains were previously described [8]. The *mycbp-RNAi* (VDRC: v41628), *y-RNAi* (v106068), *TOsk-Gal4* (v 314033), Burdock sensor [*UAS-Dcr2; NGT-Gal4; nosGal4-VP16, nos > NLS_GF’_lacZ_vas-3’UTR_burdock-target*] (v 313217) strains were from Vienna *Drosophila* Resource Center. *UAS-Dcr-2; NGT-Gal4* (BDSC: 25751) and *FRT82B/TM6C, Sb* (BDSC: 86313) were obtained from the Bloomington Stock Center. The *bam-GFP* reporter (DGRC: 118177) and *vasa-EGFP* knocked-in fly (DGRC: 118616) were from Kyoto *Drosophila* Stock Center. *hsFLP*; + ; *FRT82B*, *ubi-RFP*/*TM6B* strain was a kind gift from Dr. Wu-Min Deng (Tulane University).

### Fertility assay

Fertility assays were performed as described previously [8,63–66,69; 65]. For female fertility, five virgin females of the test genotype were crossed with three wild-type (OregonR) males in cages containing 6-cm grape juice agar plates supplemented with wet yeast paste at 25°C. Plates were replaced daily. Eggs laid on the third plate (from day 3 to day 4) were counted, and hatching was assessed after an additional 24 horus of incubation at 25°C. At least three cages per genotype were analyzed.

For male fertility, a single test male was mated with five wild-type (OregonR) virgin females in a vial at 25°C. After 3 days, the females were transferred to a new vial (vial 1), and then to new vials every 2 days for a total of four vials. Females were removed after 2 days in the fourth vial. Total progeny from all vials were counted. At least five males per genotype were tested.

### MYCBP antibody generation

Recombinant full-length MYCBP with a C-terminal HRV3Csite-6xHis tag was expressed in *E. coli* using a modified pET vector [70] and purified via Ni-sepharose (Cytiva). The His-tag was removed via HRV3C protease cleavage, and the protein was further purified on a HiTrapQ HP column (Cytiva). This antigen was used to generate rabbit polyclonal anti-sera (Pocono Rabbit Farm & Laboratory, Inc.). Rabbit polyclonal anti-MYCBP antibodies were affinity-purified using recombinant MYCBP-HRV3Csite-6xHis protein conjugated to Affigel-15 (Bio-rad).

### Immunostaining

Stereomicroscope images of dissected ovaries were taken using a Leica M125 stereomicrocsope. Ovaries from 2- to 5-day-old, yeast-fed females were dissected in 1x PBS (137 mM NaCl, 2.7 mM KCl, 10 mM Na_2_HPO_4_, 1.8 mM KH_2_PO_4_, pH 7.4) and fixed for 30 minutes at room temperature in a fix buffer (4% formaldehyde, 15 mM PIPES (pH 7.0), 80 mM KCl, 20 mM NaCl_2_, 2 mM EDTA, and 0.5 mM EGTA). Samples were washed in PBX (1x PBS + 0.1% Triton X-100) and blocked in a blocking buffer (PBX with 2% donkey serum, 3% BSA [w/v], and 0.02% NAN_3_ [w/v]) for 1 hour at room temperature. The ovaries were then incubated with primary antibodies diluted in the blocking buffer overnight at 4°C. Samples were washed three times with PBX and incubated with Alexa Fluor-conjugated secondary antibodies for 2 hours at room temperature, washed again, and mounted in VECTASHIELD PLUS antifade mounting medium with DAPI (H-2000, Vector lab). Confocal images were acquired on a Zeiss LSM700 confocal microscope at the Johns Hopkins University School of Medicine Microscope Facility.

The primary antibodies used for immunostaining were rabbit anti-MYCBP (dilution: 1/100), mouse anti-HTS (1B1) (DSHB, AB_528070, dilution: 1/100), mouse anti-Bam (DSHB, AB_10570327, 1/20), mouse anti-CycA (DSHB, AB_528188, 1/100), mouse anti-Orb (DSHB, AB_528419, 1/100), mouse anti-Sxl M18 (DSHB, AB_528464, 1/20), rabbit anti-pMad (Cell Signaling, Phospho-SMAD1/5 (Ser463/465) mAb #9516, 1/200), and rabbit anti-cleaved caspase-3 (Cell Signaling, Cleaved Caspase-3 (Asp175) #9661, 1/200), rabbit anti-Phospho-Histon H3 (Ser10) (Cell Signaling, #9701, 1/100). Secondary antibodies used were Alexa Fluor 488 Donkey anti-Mouse Igg (ThermoFisher, A21202, 1/100), Alexa Fluor 594 Donkey anti-Mouse Igg (ThermoFisher, A21203, 1/100), Alexa Fluor 594 Donkey anti-Rabbit Igg (ThermoFisher, A21207, 1/100), and Alexa Fluor 647 Donkey anti-mouse Igg (ThermoFisher, A31571, 1/100). Rhodamine phalloidin (ThermoFisher, R415, 1/100) was used to stain F-Actin.

### Germline clonal analysis

*mycbp*^*null*^ germline clones were generated using FLP/FRT-mediated recombination [33]. *mycbp*^*null*^ GSC clones were induced by heat-shocking 3-day-old females of the genotype *hs-flp*/*w*; *+ ; FRT82B, ubi-GFP/FRT82B, mycbp*^*null*^ at 37°C for 1 hour, twice daily with an 8-hour interval. Controls were *hs-flp*/*w*; + ; *FRT82B, ubi-GFP/FRT82B.* Ovaries were dissected 4, 7, and 14 days post-heat shock.

To generate *mycbp*^*null*^ mutant clones in the presence of transgenic reports, files of following genotypes were used. *hs-flp*/*w*; *otu-EGFP*/ + ; *FRT82B*, *ubi-RFP*/*FRT82B*, *mycbp*^*null*^, *hs-flp*/*w*; *otu(*Δ*Tudor)-EGFP*/ + ; *FRT82B*, *ubi-RFP*/*FRT82B*, *mycbp*^*null*^ and *hs-flp*/*w*; *sakura-EGFP*/ + ; *FRT82B*, *ubi-RFP*/*FRT82B*, *mycbp*^*null*^. Control genotypes lacking the *mycbp*^*null*^ allele were also analyzed. Flies were dissected 3–5 days after clone induction.

### Western blot

Lysates of hand-dissected ovaries and tissues were prepared by homogenizing in RIPA buffer (50 mM Tris-HCl [pH 7.4], 150 mM NaCl, 1% [v/v] IGEPAL CA-630, 0.1% [w/v] sodium dodecyl sulfate (SDS), 0.5% [w/v] sodium deoxycholate, 1 mM ethylenediaminetetraacetic acid (EDTA), 5 mM dithiothreitol, and 0.5 mM phenylmethylsulfonyl fluoride (PMSF)) [8,63,66,68]. Homogenates were centrifuged at 21,000g at 4°C for 10 min, and the protein concentrations of the supernatant were determined using the BCA protein assay kit (Pierce). Fifteen μg of total protein was loaded per lane for Western blot.

The sources and dilutions of the primary antibodies were as below. Rabbit anti-MYCBP (1/10000, generated in this study), Rabbit anti-Sakura (1/10000, [8]), rabbit anti-Otu (1/10000, [8]), mouse anti-Sxl M18 (1/1000, DSHB, AB_528464), mouse anti-Sxl M114 (1/1000, DSHB, AB_528463), rabbit anti-alpha-Tubulin [EP1332Y] (1/10000, Abcam, ab52866), mouse anti-alpha-Tubulin [12G10] (1/10000, DSHB, AB_1157911), mouse anti-FLAG (1/10000, Sigma, F1804), mouse anti-HA (1/10000, Sigma, H3663), and mouse anti-GFP [GF28R] (1/3000, Invitrogen, 14-6674-82). IRDye 800CW goat anti-mouse IgG (LiCor), IRDye 800CW goat anti-rabbit IgG (LiCor), IRDye 680RD goat anti-mouse (LiCor), and IgG IRDye 680RD goat anti-rabbit (LiCor) were used as secondary antibodies. The membranes were scanned using the Li-Cor Odyssey CLx Imaging System.

### Mass spectrometry

Immunoprecipitation of MYCBP-EGFP protein was performed using the GFP-Trap Magnetic Agarose Kit (Proteintech, gtmak-20) on dissected ovaries from flies harboring *mycbp-EGFP* transgene, with *w1118* flies as controls. Ovaries were homogenized in 200 μL ice-cold lysis buffer (10 mM Tris-HCl [pH 7.5], 150 mM NaCl, 0.5 mM EDTA, 0.05% [v/v] IGEPAL CA-630) containing 1 × protease inhibitor cocktail (100 × protease inhibitor cocktail contains 120 mg/ml 1 mM 4-(2-aminoethyl) benzene sulfonyl fluoride hydrochloride (AEBSF), 1 mg/ml aprotinin, 7 mg/ml bestatin, 1.8 mg/ml E-64, and 2.4 mg/ml leupeptin). After homogenization, the tubes were placed on ice for 30 minutes, and the homogenates were extensively pipetted every 10 minutes. The lysates were then centrifuged at 17,000x g for 10 minutes at 4°C. The supernatants were transferred to pre-chilled tubes, and 300 μL dilution buffer (10 mM Tris/Cl pH 7.5, 150 mM NaCl, 0.5 mM EDTA) supplemented with 1x protease inhibitor cocktail were added. The diluted lysates were then added to the GFP-trap magnetic beads in 1.5 mL tubes and rotated for 1 hour at 4°C. After separating the beads with a magnetic tube rack, the beads were washed three times with 500 μL wash buffer (10 mM Tris/Cl pH 7.5, 150 mM NaCl, 0.05% [v/v] IGEPAL CA-630). Proteins were eluted with 40 μL acidic elution buffer (200 mM glycine pH 2.5) followed by immediate neutralization with 5 uL neutralization buffer (1 M Tris pH 10.4).

As a quality control before mass spectrometry, ~ 5 μL of the samples were mixed with an equal volume of 2 × SDS-PAGE loading buffer (80 mM Tris-HCl [pH 6.8], 2% [w/v] SDS, 10% [v/v] glycerol, 0.0006% [w/v] bromophenol blue, 2% [v/v] 2-mercaptoethanol), heated at 95 °C for 3 min, and run on 4–20% Mini-PROTEAN TGX Precast Protein Gels (Biorad, #4561094). Silver staining was then performed by using Pierce Silver Stain Kit (ThermoFisher, 24612) to assess the quality of the immunoprecipitated protein samples. Mass spectrometry was conducted at the Mass Spectrometry Core at the Department of Biological Chemistry, Johns Hopkins School of Medicine, as previously described [8,65].

### Co-immunoprecipitation

For endogenous MYCBP co-IP, ovaries from wilt-type (*w*^*1118*^) flies were homogenized in ice-cold lysis buffer (10 mM Tris-HCl [pH 7.5], 150 mM NaCl, 0.5 mM EDTA, 0.05% [v/v] IGEPAL CA-630) containing 1 × protease inhibitor cocktail. Lysates were centrifuged at 21,000g at 4°C for 10 min, and supernatants were used for immunoprecipitation. Four μg of rabbit anti-MYCBP and control rabbit IgG (Cell Signaling, #2729) were incubated with 50 μL of Dynabeads Protein G (ThermoFisher, 10004D) for 20 min at room temperature. The beads were washed once with PBST (1x PBS with 0.1% Tween-20), then incubated with lysates at room temperature for 30 min, followed by three washes with PBST. Proteins were eluted with 2x SDS-PAGE loading buffer (80 mM Tris-HCl [pH 6.8], 2% [w/v] SDS, 10% [v/v] glycerol, 0.0006% [w/v] bromophenol blue, 2% [v/v] 2-mercaptoethanol) and heated at 70°C for 10 min. After bead separation using a magnetic tube rack, the eluted proteins in 2x SDS-PAGE loading buffer were heated again at 95°C for 3 min.

For transient expression in S2 cells, a total of 1 μg plasmid DNA (pAc5.1/V5-HisB. Invitrogen) was transfected into cells in 6-well plates using Effectene (Qiagen, 301425). After 3 days, cells were harvested and lysed in ice-cold lysis buffer with 1 × protease inhibitor cocktail, and centrifuged at 17,000x g for 10 min at 4°C. For HA-IP, supernatants were incubated with 25 μL (0.25 mg) of Pierce Anti-HA Magnetic Beads (ThermoFisher, 88837) at room temperature for 30 min. The beads were then washed three times with TBST (1x TBS [50 mM Tris/HCl and 150 mM NaCl, pH 7.6] with 0.05% Tween-20). For FLAG-IP, 2 μg of mouse anti-FLAG [Sigma, F1804] was pre-bound to 50 μL of Dynabeads Protein G (ThermoFisher, 10004D) for 10 min at room temperature. The beads were washed once with PBST. The S2 cell lysate supernatant was incubated with the washed beads at room temperature for 15 min. The beads were washed three times with PBST. In both anti-HA and anti-FLAG immunoprecipitations, proteins were eluted with 2 × SDS-PAGE loading buffer. For anti-HA, the beads in 2x SDS-PAGE loading buffer were heated at 95°C for 7 min. For anti-FLAG, the beads were heated at 70°C for 10 min, then were separated using a magnetic tube rack. The eluted proteins in 2x SDS-PAGE loading buffer were heated again at 95°C for 3 min.

### Sequential co-immunoprecipitation

S2 cells were transfected with the Sakura-EGFP-HRV3Csite-3xFLAG, EGFP-HRV3Csite-3xFLAG, MYCB-mCherry-3xHA, and Myc-Otu plasmid constructs (pAc5.1/V5-HisB. Invitrogen) with the Effectene. A total of 1 μg plasmids were transfected in each well of 6-well plates. After 3 days, cells were harvested, lysed and centrifuged as described above.

For the first IP, 2 μg of mouse anti-FLAG [Sigma, F1804] was pre-bound to 50 μL of Dynabeads Protein G and was incubated with lysate supernatant at room temperature for 15 min. Beads were washed three times with PBST, then proteins were eluted by incubation with 100 μL cleavage buffer (25 mM Tris-HCl pH 7.4, 150 mM NaCl, 5% glycerol, 2 mM EDTA) containing 25 nM GST-HRV3C protease at 4°C for 6 hours. 20 μL of the eluate was mixed with 2x SDS-PAGE loading buffer and heated at 95°C for Western blot analysis.

The remaining ~80 μL eluate was subjected to a second IP using 25 μL (0.25 mg) of Pierce Anti-HA Magnetic Beads (ThermoFisher, 88837) pre-equilibrated in lysis buffer. After a 30 min incubation at room temperature, beads were washed three times with TBST and proteins were eluted by heating at 95°C for 7 min in 2 × SDS-PAGE loading buffer.

### RT-PCR

Total RNAs from ovaries and testes and were prepared using miRVana (Thermo Fisher Scientific), followed by DNase treatment with Turbo DNase (Thermo Fisher Scientific). cDNA was synthesized from 1 μg of RNA using SuperScript VILO MasterMix (Thermo Fisher Scientific). To examine *sxl* alternative splicing, PCR was performed using GoTaq Green Master Mix (Promega) with primers *sxl*-F (5′-CTCACCTTCGATCGAGGGTGTA-3′) and *sxl*-R (5′-GATGGCAGAGAATGGGAC-3′) followed by agarose gel electrophoresis and SYBR Safe staining.

### In vitro deubiquitination assay

Purified Otu, luciferase, Sakura [8], and MCYBP proteins (latter purified as for antibody generation) were mixed with Ub-Rhodamine 110 (Ubiquitin-Proteasome Biotechnologies, M3020) in a 30 μL in reaction (20 mM Tris-HCl, pH 7.5, 200 mM NaCl, 5 mM MgCl_2_, 2 mM DTT). Reactions were performed for 60 min in a black 384-well low-volume plate and fluorescence signals were measured using SpectraMax i3x Multi-Mode microplate reader (excitation/emission: 485/20 nm and 530/20 nm).

## Supporting information

S1 Fig MYCBP expression pattern in egg chambers. (A) Confocal images of egg chambers from *mycbp-EGFP* transgenic flies. MYCBP-EGFP (green) and DAPI (blue). Scale bar: 20 μm. (B) Confocal images of egg chambers from *mycbp-EGFP* transgenic flies. MYCBP-EGFP (green), Hts (red), and DAPI (blue). Scale bar: 10 μm. (C) Confocal images of germaria from *w1118* flies. MYCBP (green) and DAPI (blue). Scale bar: 10 μm. (D) Confocal images of germaria containing control and *mycbp*^*null*^ clones. GFP (green), MYCBP (red), and DAPI (blue). Scale bar: 10 μm. Clones (which are GFP-negative) are outlined. Fly genotypes used: *hs-flp*/*w*; + ; *FRT82B*, *ubi-GFP*/*FRT82B*. *hs-flp*/*w*; + ; *FRT82B*, *ubi-GFP*/*FRT82B*, *mycbp*^*null*^.
(EPS)

S2 Fig MYCBP binds other MYCBP proteins and Myc-Otu, but not 3xMyc-CycA, Myc-Bam, 3xMyc-EGFP, or dMyc, and Sakura binds other Sakura proteins. Co-immunoprecipitation using S2 cell lysates followed by Western blotting. (A) Anti-FLAG co-immunoprecipitation to test whether MYCBP binds other MYCBP proteins. (B) Anti-HA co-immunoprecipitation to test whether MYCBP binds Otu, CycA, and Bam. (C) Anti-FLAG co-immunoprecipitation to test whether MYCBP binds dMyc. (D) Anti-FLAG co-immunoprecipitation to test whether Sakura binds other Sakura proteins.
(EPS)

S3 Fig Alphafold-predicted structures. Protein complex structures predicted using Alpha-fold. full-length MYCBP, Sakura, and human MYCBP are used while Otu(N) is 1–405 aa region and OTUD4(N) is 1–350 aa region.
(EPS)

S4 Fig In vitro deubiquitination assay. luorescence intensity representing Otu’s deubiquitinase activity in vitro using Ub-Rhodamine 110 as substrate. Mean ± SD (n = 3). Student’s t-test, unpaired, two-tailed. Firefly Luciferase was used as a negative control.
(EPS)

S5 Fig Male fertility assay. (A) Confocal images of the apical tip region of a testis from a *mycbp-EGFP* transgenic fly. MYCBP-EGFP (green), Vasa (red), and DAPI (blue). Hub cells are marked with yellow star. Scale bar: 20 μm. (B) Confocal images of the apical tip region of a testis from a control *(w1118)* fly. MYCBP (green), Vasa (red), and DAPI (blue). Hub cells are marked with yellow star. Scale bar: 10 μm. (C) Numbers of progeny obtained from crosses between test males and wild-type (OregonR) virgin females. Mean ± SD (n = 5). Student’s t-test, unpaired, two-tailed.
(EPS)

S6 Fig *mycbp*^*null*^ ovaries are tumorous. Violin plots showing the number of GSC-like cells per germaria in 2–5-day-old flies. Mean ± SD. P-value < 0.001 (Student’s t-test, unpaired, two-tailed) is indicated by ***.
(EPS)

S7 Fig *mycbp*^*null*^ germaria exhibit increased germ cell proliferation. (A) Confocal images of control (*mycbp*^*null/*^+ ) and *mycbp*^*null/null*^ ovaries and (B) control (*sakura*^*null/*^+ ) and *sakura*^*null/null*^ ovaries, stained with anti-Phospho Histone H3 (Ser10) (pH3). Hts (red), pH3 (green), and DAPI (blue). Scale bars: 20 μm.
(EPS)

S8 Fig *mycbp*^*null*^ germline clones intrinsically cause tumorous phenotypes. Number of unmarked (GFP-positive) GSC-like cells in germaria containing marked (GFP-negative) GSCs at 4, 7, and 14 days after clone induction.
(EPS)

S9 Fig *TOsk > otu*^*RNAi*^ exhibit defects in egg chambers. Confocal images of ovaries stained with phalloidin (F-Actin, red), anti-Orb (Green), and DAPI (blue). Yellow arrows indicate normal Orb localization; white arrowheads indicate cytoskeletal disorganization and dysregulation of Orb localization. Scale bar: 50 μm. *w*^*RNAi*^ served as a control.
(EPS)

S10 Fig Otu expression in germarium including in GSCs are does not depend on MYCBP and Sakura. Confocal images of germaria with control, *mycbp*^*null*^, and *sakura*^*null*^ germline clones expressing (A) Otu-EGFP or (B) Otu(ΔTudor)-EGFP. Marked clones (RFP-negative) are outlined with white and marked GSC clones are outlined with yellow. RFP (red), Otu-EGFP or Otu(ΔTudor)-EGFP (green), and DAPI (blue). Scale bar: 10 μm. Fly genotypes used: *hs-flp*/*w*; *otu-EGFP*/ + ; *FRT82B*, *ubi-RFP*/*FRT82B*. *hs-flp*/*w*; *otu-EGFP*/ + ; *FRT82B*, *ubi-RFP*/*FRT82B*, *mycbp*^*null*^. *hs-flp*/*w*; *otu-EGFP*/ + ; *FRT82B*, *ubi-RFP*/*FRT82B*, *sakura*^*null*^. *hs-flp*/*w*; *otu(ΔTudor)-EGFP*/ + ; *FRT82B*, *ubi-RFP*/*FRT82B*. *hs-flp*/*w*; *otu(ΔTudor)-EGFP*/ + ; *FRT82B*, *ubi-RFP*/*FRT82B*, *mycbp*^*null*^. *hs-flp*/*w*; *otu(ΔTudor)-EGFP*/ + ; *FRT82B*, *ubi-RFP*/*FRT82B*, *sakura*^*null*^.
(EPS)

S1 Data Numerical data underlying graphs. (XLSX)
